# PCSK9 in the Immunometabolism of Type 2 Diabetes

**DOI:** 10.3390/ijms27219723

**Published:** 2026-10-29

**Authors:** Provvidenza Villari, Antonio Maria Labate

**Affiliations:** 1 Diabetology Outpatient Service, Azienda Socio-Sanitaria Territoriale del Garda, 25024 Leno, Italy; provvidenza.villari@asst-garda.it; 2 Diabetology Outpatient Service, Azienda Socio-Sanitaria Territoriale della Franciacorta, 25032 Chiari, Italy

**Keywords:** PCSK9, type 2 diabetes, immunometabolism, inflammation, lipid trafficking, platelets, insulin secretion, cardiovascular risk

## Abstract

Proprotein convertase subtilisin/kexin type 9 (PCSK9) inhibition reduces cardiovascular events in type 2 diabetes, while experimental studies implicate PCSK9 in immune activation, platelet function and organ-specific metabolism. Whether these mechanisms contribute clinically beyond atherogenic lipoprotein lowering remains uncertain. This critical narrative review integrates an original corpus of 108 appraised publications, with targeted additions during revision. It evaluates whether findings from different compartments and experimental perturbations can be transferred to particular treatments and clinical outcomes. Mechanistic evidence implicates receptor trafficking, intracellular cholesterol, mitochondrial stress and inflammatory signalling. Effects vary by cell type and compartment: local PCSK9 promotes injury in some models but preserves pancreatic, glomerular or cardiac function in others. Global knockout, tissue-specific deletion, extracellular antibodies and hepatic silencing are therefore distinct exposures. Human biomarker and imaging studies provide translational signals but generally cannot isolate direct effects from concomitant lipoprotein changes. Randomised antibody trials establish cardiovascular efficacy and an overall reassuring glycaemic profile, without demonstrating clinically meaningful glucose lowering. PCSK9 has broader immunometabolic functions, but the available evidence does not quantify an additional clinical benefit independent of atherogenic lipoprotein lowering. Tissue-specific mechanisms and causal mediation require further investigation.

## 1. Introduction

In type 2 diabetes, the coexistence of dyslipidaemia, altered nutrient handling and chronic inflammatory activation makes the distinction between lipid and immune pathways clinically relevant. A treatment that changes the exposure of vascular cells to atherogenic particles can modify inflammatory processes without directly targeting an inflammatory mediator. Conversely, a protein originally identified through cholesterol metabolism may also influence cellular functions that are not captured by plasma low-density lipoprotein (LDL) cholesterol. Proprotein convertase subtilisin/kexin type 9 (PCSK9) provides an instructive example of both possibilities. Cardiovascular outcome trials establish the benefit of extracellular PCSK9 inhibition in patients with diabetes, whereas the experimental literature describes effects on platelet activation, immune cells, intracellular lipid handling and organ function [1,2,3,4].

Here, immunometabolism has two related meanings: metabolic regulation of immune-cell function, including changes in lipid uptake and intracellular substrate handling; and interactions between inflammatory processes and metabolic function in vascular and parenchymal tissues. We identify the relevant cell and endpoint when using the term. Associations with circulating inflammatory markers, changes in tissue lipid content and altered insulin secretion are not, by themselves, direct measurements of immune-cell metabolic reprogramming.

The central question is therefore more specific than whether PCSK9 has pleiotropic effects. It is whether the additional biology of PCSK9 supports a clinically relevant immunometabolic action of currently available inhibitors that can be distinguished from their effects on atherogenic lipoproteins. Answering this question requires separating circulating from locally produced PCSK9, extracellular neutralisation from intracellular depletion, and experimental phenotypes from clinical outcomes. The same reduction in plasma cholesterol can coexist with increased lipid accumulation in a particular tissue, and the same protein can promote injury in one cellular setting while preserving function in another [5,6,7].

This review examines these relationships in the context of type 2 diabetes. It integrates mechanistic studies with biomarker cohorts, vascular imaging, randomised trials and genetic evidence. We argue that the evidence supports a broader biological role for PCSK9 but does not yet quantify a distinct clinical benefit of PCSK9 inhibition mediated by direct immunometabolic effects independently of reductions in LDL, apolipoprotein B (apoB) and other atherogenic lipoproteins. This distinction also prevents PCSK9 inhibitors from being interpreted as glucose-lowering drugs simply because their target participates in diabetes-related pathways.

Previous reviews have already established important parts of this distinction. Päth et al. examined β-cell cholesterol homeostasis across knockout models, human variants and inhibitor-treated patients [8]. Lu et al. reviewed circulating and pancreatic PCSK9, conflicting islet findings and drugs that modify PCSK9 [9]. Katsuki et al. separated macrophage pathways by lipid and low-density lipoprotein receptor (LDLR) dependence [10]. A recent review of circulatory inflammation also distinguished direct immunomodulation from lipid-mediated plaque remodelling and cautioned against inferring systemic anti-inflammatory efficacy [11]. These concepts are therefore the starting point for this review rather than claims of conceptual priority.

Our contribution is a diabetes-focused critical synthesis that asks whether each proposed mechanism is transferable to a specific treatment and clinical endpoint. We examine the protective as well as injurious effects of local PCSK9, assess lifelong genetic exposure separately from pharmacological inhibition, and distinguish changes beyond plasma LDL cholesterol from benefit independent of atherogenic lipoproteins. The resulting interpretation connects pancreatic and organ-specific counterevidence with immune, platelet and clinical findings; it does not propose a new molecular pathway or a pooled estimate of treatment effect. Section 10 makes the overlap with earlier reviews and the contribution of the present synthesis explicit.

## 2. Literature Search and Critical Appraisal

This is a critical narrative review informed by structured searches of PubMed, Embase, Web of Science and the Cochrane Central Register of Controlled Trials (CENTRAL) and by supplementary retrieval of relevant publications. The evidence assembled for appraisal covered PCSK9 and its inhibition in relation to diabetes, glucose metabolism, inflammation, immune and vascular cells, platelet function and organ-specific lipid handling. The purpose was to reconcile mechanistic and clinical findings rather than estimate a pooled treatment effect. The initial appraisal comprised 108 publications: 98 from a full-text retrieval list of 113 records and 10 additional publications. These publications do not represent 108 independent cohorts; several report secondary analyses or pooled data from the same trials.

An evidence inventory reconciles the appraised corpus with the bibliography (Supplementary File S2). The submitted version cited 68 of the 108 publications. During editorial revision, all 40 appraised but uncited publications were reassessed against the manuscript scope and claims; 17 were integrated and 23 retained in the inventory without individual citation. Thus, 85 publications from the original corpus are cited. Targeted additions comprised Schmidt et al., Ference et al. and Peyot et al., plus four contextual reviews, bringing the bibliography to 92 references before external peer review. The main texts of Schmidt et al. and Ference et al. were verified; Peyot et al. remained abstract-only and is used as contextual counterevidence. Separate external supplements for these additions were not appraised. During peer review, AMUNDSEN and the FOURIER inflammatory-risk analysis were added, giving 94 references. These two additions and their access levels are recorded separately in Supplementary File S2. The original 108-publication denominator and the decisions on the 40 initially uncited records remain unchanged and separately traceable.

Fifteen articles in the retrieval list could not be obtained in full text. Lack of access was recorded separately from scientific ineligibility, and findings available only through abstracts were not used as decisive evidence. The accessible main texts were examined for study design, population or model, intervention, comparator, outcomes and limitations. External Supplementary Files that were unavailable were not treated as verified material. The synthesis incorporated previously completed critical records alongside newly examined full texts.

Appraisal focused on the identity of the intervention and the validity of the proposed causal interpretation. Particular attention was paid to global versus tissue-specific knockout, extracellular antibodies versus hepatic silencing, recombinant protein concentrations, lipid-matched comparisons, rescue experiments, sample size, independence of biological replicates and overlap between clinical datasets. In observational studies, adjustment for LDL cholesterol was distinguished from demonstration of an LDL-independent mechanism. In subgroup analyses, differences in statistical significance were not interpreted as treatment interactions without an appropriate interaction test.

No new meta-analysis, formal Grading of Recommendations Assessment, Development and Evaluation (GRADE) assessment or fully reproducible systematic-review selection process was performed. The review does not claim exhaustive coverage of all records retrieved in the initial searches. Its conclusions apply to the critically appraised corpus, with particular caution where inaccessible supplements, incomplete reporting or conflicting results prevent definitive interpretation.

### Use of Generative Artificial Intelligence and Preparation of Illustrations

ChatGPT in the Codex environment (OpenAI, San Francisco, CA, USA; accessed October 2026; the model and software version identifiers used for the original preparation were not retained) assisted with literature organisation, appraisal support, drafting and document preparation. The authors directed the review and remain responsible for source verification and interpretation. Earlier submitted versions of Figure 1 and the graphical abstract used OpenAI image generation. For this revision, both illustrations were rebuilt as deterministic vector schematics using AI-assisted Python code and geometric primitives; text was placed programmatically. The Python version used for the original illustration build was not recorded. No published figure, third-party illustration or experimental image was used as an artwork asset in the rebuilt illustrations. Original technical prompts for the earlier raster images were not retained and have not been retrospectively reconstructed. Supplementary File S3 documents this limitation, the revised design specifications and the reproducible illustration source code.

## 3. PCSK9 Compartments and the Meaning of Effects Beyond LDL

### 3.1. Circulating and Intracellular PCSK9 Are Different Exposures

The familiar therapeutic model concerns liver-derived PCSK9, LDL receptor availability and the clearance of circulating LDL particles. Experimental work extends this model to other receptors and tissues. PCSK9 can regulate cluster of differentiation 36 (CD36) in adipocytes and liver and influence very-low-density lipoprotein receptor (VLDLR)-related adipose phenotypes. These observations show that PCSK9 biology extends beyond plasma LDL cholesterol, but it remains part of lipid biology: fatty-acid uptake, intracellular cholesterol and receptor trafficking can change even when circulating LDL is unchanged [5,12].

A further distinction concerns the location of the relevant PCSK9 pool. Extracellular antibodies neutralise accessible PCSK9; they do not reproduce deletion of the gene in every cell. Inclisiran reduces hepatic PCSK9 production and likewise does not recreate global lifelong deficiency. Experiments involving pancreatic or cardiomyocyte PCSK9 therefore answer questions about local biology that cannot automatically be transferred to either treatment. The separation is supported by mouse studies in which hepatic deletion lowers cholesterol without reproducing the glucose phenotype of global deletion and by cardiac experiments in which secreted PCSK9 does not rescue the consequences of intracellular depletion [7,13]. Local production does not by itself identify the site of action: PCSK9 synthesised within a tissue may be secreted and act extracellularly through autocrine or paracrine routes, whereas intracellular functions involve a distinct pool. Figure 1 separates these source and action dimensions alongside the tissue-specific processes discussed in this review.

### 3.2. Three Claims Require Three Different Kinds of Evidence

An effect beyond plasma LDL cholesterol may still depend on intracellular lipid accumulation. An effect independent of LDLR may involve another lipid receptor. A clinically meaningful non-lipid benefit requires an additional step: evidence that an intervention improves a relevant outcome through a pathway separable from changes in atherogenic particles. These claims are not interchangeable. For example, PCSK9-dependent CD36 signalling in washed platelets is stronger evidence for a direct cellular effect than an observational association between circulating PCSK9 and an inflammatory marker. Neither result alone establishes the proportion of clinical cardiovascular benefit attributable to that pathway [3,14].

Statistical adjustment cannot fully substitute for this biological separation. LDL measurements are imperfect proxies for cumulative exposure and do not capture apoB particle number, lipoprotein composition or tissue uptake. Treatment-induced changes in LDL, lipoprotein(a) (Lp(a)) and inflammatory imaging may also occur together. A residual association after adjustment can identify a hypothesis but does not provide the same evidence as receptor perturbation, rescue or a comparator producing equivalent lipoprotein exposure. Table 1 summarises the implications of these distinctions.

## 4. Immune Cells and Vascular Signalling

### 4.1. Macrophage Activation Depends on the Stimulus and Receptor Pathway

Macrophages provide substantial experimental evidence linking PCSK9 to inflammatory activation. Recombinant PCSK9 increases cytokine expression and nuclear factor κB (NF-κB)-related responses in cultured macrophages. However, attenuation in LDLR-deficient cells indicates that much of the response in the studied system depends on LDLR. This finding does not eliminate a contribution from intracellular lipid handling, and recombinant protein concentrations and short exposure periods limit comparison with chronic human disease [15].

PCSK9 also regulates scavenger receptors and oxidised LDL uptake. In tumour necrosis factor α (TNF-α)-primed murine macrophages, PCSK9 increases lectin-like oxidised low-density lipoprotein receptor 1 (LOX-1), scavenger receptor class A (SR-A) and CD36-related responses, while knockout or silencing reduces receptor expression and oxidised LDL uptake. This circuit connects inflammation with lipid uptake rather than cleanly separating them. Its interpretation must retain the experimental context: activated murine macrophages, specific lipid substrates and limited numbers of independent experiments [20].

In apolipoprotein E (ApoE)-deficient mice, lentiviral PCSK9 silencing reduced atherosclerotic lesions, macrophage accumulation and Toll-like receptor 4 (TLR4)/NF-κB-related signals without statistically significant differences in measured plasma lipids. This adds in vivo support to the cellular observations but does not demonstrate equivalent lipid exposure: several molecular endpoints had small samples, the main control received saline, and a vector-control experiment was confined to an unavailable supplement. Systemic lentiviral silencing also differs from extracellular antibody treatment [21].

Apparently opposite results become more coherent when the lipoprotein substrate is considered. PCSK9 secreted by vascular smooth muscle cells reduces macrophage LDLR and uptake of native LDL or β-very-low-density lipoprotein (VLDL). This does not directly contradict enhanced oxidised LDL uptake through scavenger receptors. The two observations involve different receptors, stimuli and lipoprotein routes. Describing PCSK9 as uniformly increasing macrophage lipid accumulation would therefore obscure the actual evidence [22].

### 4.2. Dendritic Cells and Lymphocytes Support Local Immunometabolic Interactions

Studies of human dendritic cells and T cells show that oxidised LDL can induce PCSK9 together with maturation markers and inflammatory activity. PCSK9 silencing attenuates several dendritic-cell responses and modifies subsequent T-cell activation. These experiments support a local role at the interface between lipid exposure and adaptive immunity. They do not demonstrate that a circulating antibody reaches the same intracellular pool or reproduces the effect of silencing in dendritic cells [16].

The reported inability of exogenous PCSK9 to restore some silencing phenotypes is compatible with an intracellular contribution, although the relevant supplementary experiments were not independently available in the appraised material. This makes the compartment question particularly important. A biological effect of local PCSK9 depletion should not be labelled a clinical class effect of antibodies or hepatic siRNA without evidence linking exposure to the relevant cells and demonstrating target engagement.

Human observations provide a more limited bridge. In a stable coronary disease cohort, PCSK9 was associated with the relative proportions of classical and non-classical monocytes, mainly among statin-treated participants. Only 69 patients had both PCSK9 and flow-cytometry measurements, circulating cytokines were not consistently associated, and multiple comparisons and the compositional nature of monocyte percentages restrict interpretation. These findings support an exploratory association with immune-cell distribution, not evidence that PCSK9 inhibition changes monocyte function or improves clinical outcomes [23].

### 4.3. PCSK9 Can Be Downstream of Inflammation

The direction of the relationship is not invariably one of PCSK9 to inflammation. Experimental activation of the NOD-like receptor family pyrin domain containing 3 (NLRP3) inflammasome and interleukin 1β (IL-1β) signalling increases PCSK9 production and secretion. In that system, deletion of PCSK9 has little effect on upstream NLRP3 or IL-1β expression. The dominant direction is therefore one of inflammation to PCSK9 under the tested conditions, rather than a self-sustaining loop established in both directions [24].

Human experimental inflammation provides a further qualification. In a controlled endotoxin study, inflammatory markers rose while PCSK9 did not differ significantly from the placebo. This small study in participants without diabetes does not exclude a role in chronic inflammation or severe infection, but it argues against using circulating PCSK9 as an obligatory marker of systemic inflammatory activation [25].

### 4.4. Vascular Cells and Extracellular Vesicles Broaden the Model

Vascular smooth muscle cells can connect haemodynamic or metabolic stress to local PCSK9 expression. Studies of altered flow, high glucose and insulin resistance support associations with inflammatory signalling and oxidative stress. Yet changes in signalling are not equivalent to the reversal of a cellular phenotype: in one study, PCSK9 inhibitors reduced some oxidative or signalling readouts without significantly reducing the enhanced proliferation and migration of cells from obese Zucker rats [26,27].

Extracellular vesicles from PCSK9-overexpressing smooth muscle cells promote inflammatory and metabolic responses in recipient cells. However, overexpression also changes other vesicle proteins and microRNAs. Without selective rescue or neutralisation, the observed vesicle phenotype cannot be attributed exclusively to PCSK9 cargo. These experiments establish a plausible communication route while leaving the identity of the decisive mediators unresolved [28].

Endothelial responses are similarly conditional. Experimental PCSK9 can limit LDLR-mediated uptake of lipopolysaccharides and attenuate some downstream endothelial responses. This protective direction contrasts with studies associating PCSK9 with oxidative injury or hypoxia-related cell death. Together, the findings support a context-dependent model rather than a universally pro-inflammatory function [29,30].

A high-glucose microvascular endothelial model adds a different perturbation: PCSK9 silencing reduced LOX-1-related oxidative, lipid and inflammatory responses, and LOX-1 overexpression attenuated those effects. The rescue supports involvement of this pathway in the tested cells. The accompanying small human comparison was age-imbalanced and reported unusually high PCSK9 concentrations; it should not be treated as validation of the cellular mechanism. Missing osmotic controls, uncertain replicate independence and the absence of a therapeutic antibody experiment further limit translation [31].

## 5. Platelet Thrombosis and Vascular Imaging

### 5.1. Direct Platelet Effects Provide a Comparatively Strong Mechanistic Signal

The platelet literature contains some of the most specific evidence for a direct action of extracellular PCSK9. Qi and colleagues combined washed platelet experiments, CD36 perturbation, LDLR-deficient models and neutralisation with evolocumab. PCSK9 enhanced agonist-induced activation and thrombosis-related responses through CD36-associated signalling, oxidative stress and downstream kinase pathways. The design is more informative about a direct cellular mechanism than correlations between plasma PCSK9 and platelet markers [3].

Human studies relating PCSK9 to CD36-associated oxidative platelet activation are consistent with this mechanism but do not establish a biomarker-guided treatment strategy. PCSK9 concentration, acute coronary disease, antiplatelet treatment and metabolic status may be interrelated. In a ST-elevation myocardial infarction (STEMI) cohort, associations with inflammation, platelet markers and recurrent events varied with diabetes and the analytical threshold; predictive improvement was not externally validated [14,32].

A small before-and-after study of PCSK9 inhibition reported reductions in some platelet responses, with signals in participants with diabetes. The absence of a randomised comparator, attrition and multiple subgroup analyses restrict causal interpretation. A significant change in the diabetes group alongside a non-significant change in the non-diabetes group does not establish a diabetes-specific interaction. The mechanism remains plausible, while its contribution to clinical event reduction is unquantified [33].

### 5.2. Imaging Suggests Local Benefit but Does Not Isolate Its Mediator

A prespecified PACMAN-AMI sub-study provides a randomised imaging signal. Twenty participants were analysed after allocation to alirocumab or the placebo, both with rosuvastatin. The reported between-group difference in the percentage change of carotid fluorodeoxyglucose positron emission tomography (FDG-PET) target-to-background ratio was −27.7%, following the notation in the original report. The result supports a treatment effect on a local vascular surrogate. LDL cholesterol also fell more with alirocumab, and the small sample does not allow lack of correlation between changes to establish mechanistic independence [34].

Coronary plaque imaging and transcriptomic studies provide related observations. In the diabetes analysis of YELLOW III, evolocumab-associated plaque changes were observed in both diabetes and non-diabetes groups. However, post hoc subgroup comparisons and the absence of a placebo group prevent attribution of the changes to a distinct immunometabolic pathway or demonstration of enhanced responsiveness in diabetes [35].

In a serial coronary computed tomography (CT) study in type 2 diabetes, evolocumab treatment was associated with lower pericoronary adipose attenuation and concurrent changes in LDL and Lp(a). Statistical mediation suggested a relationship with Lp(a), but neither treatment assignment nor the individual biomarker changes were independently randomised. The result is useful for hypothesis generation and should not be described as proof of a non-lipid anti-inflammatory action [36].

Functional vascular measurements show a related signal. In an uncontrolled pilot involving 30 participants with human immunodeficiency virus (HIV) or dyslipidaemia, almost all without diabetes, evolocumab treatment was followed by improved coronary endothelial responses despite no significant change in several systemic inflammatory markers. The marked concurrent LDL reduction, selection for abnormal baseline endothelial function and absence of a placebo prevent attribution to a direct anti-inflammatory pathway. Lack of correlation between LDL change and the functional response in this small sample does not establish independence [37].

### 5.3. Systemic Markers Do Not Establish a Class-Wide Anti-Inflammatory Effect

In a prospective study of 89 treated patients, including 14 with diabetes, lipoprotein-associated phospholipase A2 (LpPLA2) decreased, and its change was strongly associated with the LDL change. Most cytokines, high-sensitivity C-reactive protein (hsCRP) and measures of high-density lipoprotein (HDL) antioxidant function did not improve significantly. Isolated exploratory cytokine findings in an uncontrolled comparison do not establish systemic anti-inflammatory efficacy. Similarly, variance unexplained by LDL in a regression model cannot be assigned to a pleiotropic mechanism [38].

Not all human findings are null. A single-centre study reported as randomised compared evolocumab with simvastatin in 140 patients with type 2 diabetes and reported lower concentrations of several inflammatory markers after treatment. LDL lowering differed between groups, allocation concealment and blinding were not adequately described, and the appraisal identified inconsistencies in units and statistical reporting. The favourable signal merits consideration but cannot establish a direct anti-inflammatory effect independent of lipoprotein lowering or a glucose-lowering benefit [39].

Human transcriptional findings are also mixed. A study of coronary disease with very high Lp(a) reported unchanged interleukin-1β (IL1B) and interferon-γ (IFNG) gene expression, increased tissue factor (F3) and vascular endothelial growth factor A (VEGFA) and reduced SERPINE expression during antibody treatment. Although the parent cohort included placebo allocation, the reported main gene-expression comparison was predominantly within treated participants. Whole-blood messenger RNA (mRNA) does not establish tissue-specific protein activity, and the findings do not support a uniform anti-inflammatory or antithrombotic interpretation [40].

SPIRE analyses show that residual inflammatory risk persists despite marked LDL lowering with bococizumab. Elevated hsCRP identifies higher subsequent event rates, but hsCRP categories were not randomised and the treatment did not significantly reduce hsCRP. The study supports the coexistence of lipid and inflammatory risk; it does not establish that PCSK9 is the cause of the remaining inflammation or that its inhibition resolves it. Early trial termination and antibody immunogenicity also limit direct generalisation to fully human antibodies [41].

The FOURIER analysis of participants with autoimmune or inflammatory diseases illustrates a separate distinction. Evolocumab reduced cardiovascular events while hsCRP remained unchanged. The subgroup was small and heterogeneous, the analysis was post hoc, and interaction testing did not conclusively establish differential benefit for the primary endpoint. The result is compatible with effective lipid lowering in a high-risk inflammatory setting; it does not show that suppressing inflammation mediated the benefit [42].

## 6. Glucose Homeostasis and the Pancreatic Compartment

### 6.1. Local Cholesterol Trafficking Can Alter Insulin Secretion

Global and tissue-directed PCSK9 deletion studies identify a relationship between pancreatic receptor trafficking, intracellular cholesterol and insulin secretion. In the global knockout model examined by Da Dalt and colleagues, glucose intolerance and reduced circulating insulin coexist with increased pancreatic insulin content and islet cholesterol accumulation. Rescue through concomitant LDLR deletion supports involvement of the LDLR pathway. Hepatic deletion lowers circulating cholesterol without reproducing the pancreatic phenotype, separating plasma PCSK9 reduction from loss of local pancreatic function [13].

Pancreatic deletion experiments further connect PCSK9 to secretory-pathway organisation. Nevertheless, developmental pancreatic deletion is not equivalent to inducible deletion confined to adult β-cells. Effects on intracellular cholesterol, vesicle trafficking and insulin secretion must be interpreted within the actual genetic model. These experiments show how a phenotype can be beyond plasma LDL while remaining dependent on cellular lipid metabolism [4].

The phenotype is not uniform across studies. Early work in young PCSK9-deficient mice reported preserved insulin secretion and glucose tolerance, whereas other studies found islet abnormalities and impaired glucose handling at older ages. Sex and diet modify the results, and some broad statements in abstracts are stronger than individual comparisons in the tables. The discordance is biologically relevant: it limits claims that PCSK9 loss invariably causes β-cell failure [43,44,45].

Peyot and colleagues reported preserved glucose tolerance, insulin release and ex vivo glucose-stimulated insulin secretion after substantial β-cell-specific PCSK9 inactivation, as well as in their whole-body knockout model. The β-cell phenotype is therefore not uniformly reproduced across genetic experiments. For this publication, only the abstract was available for verification in the present revision; the reported null phenotype is included as contextual counterevidence, not as a definitive assessment of the experimental methods or proof of long-term safety. It should be considered alongside the full-text evidence for pancreatic cholesterol handling and the differences between genetic depletion and extracellular treatment [19].

### 6.2. Human Cellular Findings Do Not Reproduce a Universal Secretion Defect

Experiments in human β-cell models demonstrate the effects of PCSK9 modulation on LDLR and LDL uptake but do not show a consistent significant change in glucose-stimulated insulin secretion after short exposures. The limited number of islet donors, partial knockdown and the use of an immortalised cell line prevent a null result from proving long-term equivalence. Conversely, these results do not support assuming that extracellular PCSK9 neutralisation reproduces severe pancreatic dysfunction from global mouse deletion [46].

The distinction between biological involvement and therapeutic glucose lowering is essential. A protein can participate in insulin secretion or insulin resistance without its inhibition being an effective diabetes treatment. PCSK9 inhibitors should therefore be discussed as lipid-lowering therapies with broader mechanistic possibilities rather than placed in the same therapeutic category as incretin-based glucose-lowering agents.

### 6.3. Nutritional and Insulin Regulation of PCSK9 Is Context Dependent

Insulin and nutritional signalling can regulate PCSK9 expression, but the direction is not consistent across models. Primary hepatocyte and rodent experiments support insulin-dependent induction under some conditions, with responses modified by fasting, hepatic insulin receptor status and other hormonal pathways. These interventions affect several components of lipid metabolism and do not isolate the consequences of pharmacological PCSK9 inhibition [47,48].

A human clamp study in postmenopausal women with overweight or obesity instead reported a fall in circulating PCSK9 during insulin administration. The participants did not have diabetes, and the absence of a time-matched fasting control leaves diurnal and fasting effects unresolved. The result nevertheless cautions against extrapolating a universal insulin-to-PCSK9 direction from rodents to humans [49].

Glucose-responsive transcription provides another route to PCSK9 regulation. Cell and mouse experiments with metformin implicated carbohydrate-responsive element-binding protein (ChREBP), supported by promoter perturbation, binding assays and interventions that opposed the reduction in PCSK9 expression. This pathway is distinct from a universal insulin-driven response. However, high experimental exposures, unavailable supplementary methods and a small uncontrolled human component outside type 2 diabetes limit clinical inference. The study does not establish that PCSK9 mediates the clinical benefits of metformin or that PCSK9 inhibition reproduces glucose-lowering therapy [50].

Experimental links between statin-associated adipose insulin resistance, geranylgeranyl pyrophosphate depletion and PCSK9 add another possible pathway. These findings arise from cultured adipose tissue and genetically modified mice, with signalling endpoints rather than human glucose outcomes. They cannot demonstrate that prescribing a PCSK9 antibody reverses statin-related diabetes risk [51].

## 7. Kidney Liver and Cardiac Tissue

### 7.1. Kidney Studies Reveal Opposing Compartment-Specific Effects

Experimental diabetic nephropathy studies support both injurious and protective roles. In a tubular model, PCSK9 inhibition was associated with reduced mitochondrial deoxyribonucleic acid (DNA)-related cyclic GMP–AMP synthase (cGAS)/stimulator of interferon genes (STING) activity, inflammation and renal injury. Lipid concentrations also decreased in vivo, and the main text did not clearly identify the antibody sufficiently to establish equivalence with a marketed product. These results support a candidate tubular pathway, while leaving lipid independence and clinical transferability unresolved [52].

A separate study reported reduced glomerular PCSK9 in diabetic kidney disease and greater renal lipid accumulation, podocyte injury and albuminuria after PCSK9 deletion. The coexistence of lower circulating cholesterol with worse renal lipid deposition is particularly informative: plasma cholesterol cannot serve as a complete surrogate for tissue lipid exposure. Cell experiments point in the same protective direction for local PCSK9, but biopsies remain observational and the global knockout does not reproduce extracellular antibody therapy [6].

The ZSF1 rat study of alirocumab did not demonstrate renoprotection and reported worse values for some renal outcomes. It also failed to reproduce the expected renal protection of empagliflozin within the model, which restricts clinical transferability. The combined kidney literature therefore supports compartmental heterogeneity rather than a conclusion that PCSK9 inhibitors are either established renal protective drugs or nephrotoxic treatments in human type 2 diabetes [53].

### 7.2. Hepatic Triglyceride Storage and Cholesterol Injury Must Be Separated

PCSK9-dependent regulation of CD36 can alter fatty-acid uptake and hepatic triglyceride storage. In one experimental setting, PCSK9 deficiency increases hepatic CD36 and triglyceride accumulation. This demonstrates a receptor and substrate effect; it does not by itself establish clinically important liver injury after treatment with an antibody [5].

Models of steatohepatitis show more complex and sometimes conflicting responses. Silencing or deletion can reduce inflammation in some settings, whereas lipid accumulation does not consistently change in the same direction. Studies involving yes-associated protein (YAP)-related signalling and different dietary models illustrate how steatosis, inflammation and receptor expression can diverge. Internal reporting inconsistencies and unavailable supplementary methods require caution when assigning a uniform pathway [54,55].

Cholesterol-enriched diets in globally deficient mice provide an additional counterexample. PCSK9 deletion can increase hepatic free cholesterol, inflammation and fibrosis, even while triglyceride content falls. The distinction between triglyceride storage and free-cholesterol toxicity is mechanistically important, but the global knockout, dietary exposure and small experimental samples cannot be interpreted as evidence of liver toxicity from currently used inhibitors [56].

Human biopsy observations also show that PCSK9 mRNA, tissue protein and circulating concentrations need not move together. In a bariatric-surgery cohort, the hepatic mRNA signal differed from the immunohistochemical protein signal, while circulating PCSK9 did not reliably distinguish histological severity. Observational design and reporting inconsistencies preclude a validated prognostic or treatment-selection role. PCSK9 inhibition is not established as a therapy for metabolic dysfunction-associated steatotic liver disease on the basis of these findings [57].

### 7.3. Cardiac Intracellular PCSK9 Differs from a Circulating Therapeutic Target

Cardiomyocyte-specific deficiency compromises mitochondrial respiration, alters mitochondrial membrane cholesterol and produces cardiac dysfunction in mice without major changes in circulating lipids. Failure of secreted PCSK9 to rescue the cellular defect supports an intracellular function. These findings are strong evidence that local PCSK9 biology can differ from the familiar hepatic pathway; they are not evidence that extracellular neutralising antibodies produce the same cardiac injury [7].

Other experiments report reduced cardiac fibrosis or improved diabetic cardiomyopathy after alirocumab, with changes in inflammatory, oxidative and kinase pathways. However, concurrent reductions in LDL or glucose, incomplete pathway perturbation and small samples limit attribution to a direct immunometabolic mechanism. Similar total cholesterol values or a non-significant difference between lipid-lowering regimens do not establish equivalent lipoprotein exposure [58,59].

Immune and cardiomyocyte pathways also need separation. In a global-knockout myocardial infarction model, macrophage depletion abolished the functional advantage associated with PCSK9 loss, supporting involvement of the immune compartment. The brief follow-up, small samples and absence of measured lipids prevent a conclusion about chronic remodelling, lipid independence or antibody efficacy [60]. A separate study linked PCSK9 to mitochondrial DNA damage, reactive oxygen species, NLRP3-related signalling and cardiomyocyte pyroptosis using several downstream perturbations. High recombinant-protein exposures, global knockout and a small confounded human comparison again limit extrapolation [61]. Together with the cardiomyocyte-specific deficiency findings, these studies argue for identifying the manipulated pool and injury context rather than assigning one direction of cardiac effect to all PCSK9 interventions [7].

Small-molecule experimental inhibitors also require separate treatment. Studies using SBC-115076 report improved mitochondrial or metabolic measures in insulin-resistant rats, but the compound is not an extracellular antibody or hepatic siRNA. Changes in body weight and LDL accompany the experimental effects, and specificity or rescue evidence is incomplete. These results cannot be used to claim clinical superiority over statins or an established glucose-lowering effect of the marketed PCSK9 inhibitor class [62,63].

## 8. Circulating Biomarkers and Genetic Evidence

### 8.1. A Circulating Concentration Is Not a Universal Immunometabolic Biomarker

Associations between PCSK9 and glycated haemoglobin (HbA1c), insulin resistance, renal function or cardiovascular events vary across populations. A study in patients undergoing coronary angiography found an adjusted association with HbA1c in the diabetes group, but its cross-sectional design cannot establish whether PCSK9 worsens glycaemia, reflects metabolic disease or is influenced by treatment. Differences in subgroup significance likewise do not prove effect modification [64].

The relation to glycaemic control is not directionally uniform. In a selected cohort of 116 patients with type 2 diabetes, PCSK9 correlated with pentraxin 3 and several apolipoproteins, while HbA1c was inversely associated in the reported multivariable model. Selection for poor glycaemic control, stepwise modelling and partly previously described clinical data limit the result. This adds an inflammatory correlate and a counterpoint to the positive HbA1c association, rather than a validated prognostic test [65].

Age and sex may also condition these associations. A cross-sectional study of 286 young participants found higher PCSK9 with obesity or type 2 diabetes mainly among women. Pubertal and hormonal factors were incompletely characterised, and significance in one sex but not the other does not establish interaction without a corresponding test. These data support population-specific interpretation rather than a universal circulating PCSK9 phenotype [66].

Prospective findings are not uniformly positive. The association between PCSK9 and cardiovascular events observed in DIABHYCAR was not replicated in SURDIAGENE. Differences in background statin and insulin use, outcome definitions and population risk may contribute to the results. The evidence therefore does not support a single circulating threshold that identifies immunometabolic risk or selects patients for PCSK9 inhibition [67].

A larger angiography-based cohort provides a positive prognostic finding: among 2984 patients with type 2 diabetes, higher PCSK9 was associated with subsequent cardiovascular events after adjustment. Selection for angiography, incomplete characterisation of treatment changes and residual confounding limit generalisability and causal interpretation. Considered alongside the discordant DIABHYCAR and SURDIAGENE findings, this strengthens the case for heterogeneity rather than a single universally predictive threshold [68].

In IT-DIAB and ELSA-Brasil, circulating PCSK9 was not significantly associated with conversion to type 2 diabetes in the main analyses. Secondary associations with glycaemic deterioration were modest and do not override the primary null findings. A lifestyle study likewise found substantial metabolic improvement with only a small, non-significant PCSK9 change. These observations weaken a model in which circulating PCSK9 tracks metabolic dysfunction consistently across clinical settings [69,70].

Renal biomarker data are also heterogeneous. A diabetic kidney disease cohort did not show a clear relation of circulating PCSK9 to estimated glomerular filtration rate (eGFR) category or albuminuria, while treatment combinations and fasting duration influenced measured concentrations. Together with the opposing local renal experiments, this argues against interpreting a plasma concentration as a direct measure of renal PCSK9 activity [71].

Conversely, another study in 145 patients with type 2 diabetes and 37 controls found higher circulating PCSK9 associated with albuminuria and lower eGFR, alongside increased expression in experimental diabetic models. Its cross-sectional design, modest discrimination and covariate-selection methods leave temporal direction and confounding unresolved. The positive association therefore complements, rather than negates, the null renal biomarker findings; neither establishes that lowering circulating PCSK9 protects the kidney [72].

### 8.2. Genetic Proxies Do Not Recreate a Single Drug Exposure

PCSK9 genetic evidence must be interpreted according to the variant and instrument. A loss-of-function C679X study, an R46L glucose-homeostasis study and multi-variant Mendelian randomisation analyses do not provide interchangeable exposures. Lifelong effects, ancestry, magnitude of lipid change and pleiotropy differ. Findings suggesting greater diabetes risk in one genetic framework do not automatically predict the effect of an extracellular antibody initiated in adulthood [73,74,75].

Large target-based genetic analyses also reported a diabetes-risk signal. Schmidt and colleagues combined four PCSK9 variants using data from more than 550,000 individuals, including 51,623 diabetes cases. Scaled to 1 mmol/L genetically lower LDL cholesterol, the combined estimate was an odds ratio of 1.29 (95% confidence interval (CI) 1.11–1.50), with higher fasting glucose and bodyweight. The analysis combined incident and prevalent diabetes, was restricted to European ancestry and estimated lifelong genetic exposure; it did not supply the absolute risk or treatment effect of an antibody started in adulthood [76].

Ference and colleagues compared seven-variant PCSK9 and six-variant 3-hydroxy-3-methylglutaryl-coenzyme A reductase (HMGCR) genetic scores in 112,772 participants from 14 studies. Per 10 mg/dL genetically lower LDL cholesterol, the PCSK9 score was associated with lower odds of myocardial infarction or coronary death (odds ratio (OR) 0.81, 95% CI 0.74–0.89) and higher diabetes odds (OR 1.11, 1.04–1.19), similar to HMGCR. The primary diabetes analysis combined 4340 prevalent and 6295 incident cases. In the prospective fasting-glucose subgroup, the PCSK9 estimate for incident diabetes was 1.22 (1.03–1.45) with impaired fasting glucose and 0.99 (0.84–1.17) with normal fasting glucose; these subgroup estimates should not establish an exclusive susceptible group without appropriate interaction evidence. Lifelong genetic exposure and LDL-based scaling cannot quantify the effect of an antibody started in adulthood. The authors themselves noted that extracellular antibody binding may differ biologically from the genetic exposure. The main text, figures and table were verified for this revision; the separate supplementary appendix to that cited publication was not available [77].

A multiomic Mendelian randomisation study across five populations found no consistent robust PCSK9-associated diabetes effect across its principal analyses. Some estimates were imprecise, an ancestry-specific nominal signal did not survive the study threshold and co-localisation support was incomplete. The overall literature is therefore better described as heterogeneous than as either uniformly reassuring or uniformly diabetogenic [78].

Genetic estimates should complement, rather than replace, randomised pharmacological evidence. A genetic meta-analysis may reuse populations already represented in the underlying studies; its sample totals should not be counted as entirely new independent participants. Scaling an estimate to a unit of genetically lower LDL also does not prove that the biological exposure is equivalent to the dose or duration of a clinical inhibitor.

## 9. Clinical Treatment Evidence in Type 2 Diabetes

### 9.1. Cardiovascular Benefit of Antibodies Is Established

The strongest evidence comes from prespecified diabetes analyses of FOURIER and ODYSSEY OUTCOMES. Evolocumab reduced the primary cardiovascular endpoint in participants with diabetes, with a hazard ratio of 0.83. In ODYSSEY OUTCOMES, the corresponding hazard ratio with alirocumab was 0.84. Relative benefit did not show convincing heterogeneity according to glycaemic status, while higher baseline risk in diabetes produced greater absolute benefit [1,2].

These randomised comparisons establish the effect of adding the antibody to background therapy. They do not separately randomise the LDL response, Lp(a) response and direct cellular effects. The event reduction therefore cannot be partitioned confidently into lipid-mediated and immunometabolic components from the subgroup results alone. Demonstrated cardiovascular efficacy and an unquantified additional mechanism can coexist without weakening the clinical indication.

AMUNDSEN tested evolocumab initiated before percutaneous coronary intervention for acute myocardial infarction. Low-density lipoprotein cholesterol (LDL-C) target attainment improved, but all-cause death or unplanned cardiovascular hospitalisation at 12 months did not differ significantly from standard care (14.6% versus 15.4%; adjusted odds ratio 0.94, 95% confidence interval 0.73–1.19). The result argues against assuming an early clinically meaningful pleiotropic benefit in this setting; it does not exclude every biological mechanism or later benefit. AMUNDSEN was not restricted to type 2 diabetes [79].

### 9.2. Glycaemic Safety Does Not Imply Glucose-Lowering Efficacy

The major trial analyses did not show a significant overall increase in incident diabetes: the reported hazard ratio was 1.05 with evolocumab in FOURIER and 1.00 with alirocumab in ODYSSEY OUTCOMES. These results support an overall reassuring glycaemic profile during the studied follow-up. They do not exclude very late effects or establish improvement in glucose control [1,2].

Pooled ODYSSEY trials and the BANTING study are consistent with the absence of a substantial short-term glucose-lowering effect. In BANTING, median HbA1c changes were similar in both groups despite marked LDL reduction. Small differences in HbA1c in larger datasets may reach statistical significance without representing clinically meaningful diabetes treatment. Treatment changes in observational cohorts further complicate the interpretation of stable average HbA1c [80,81,82].

An observational analysis suggesting lower incident diabetes with PCSK9 inhibitors has important selection and exposure-timing limitations, including conditioning on survival and residual differences after matching. Such findings cannot establish an antidiabetic effect or displace the randomised evidence. Pharmacovigilance reports of hyperglycaemia likewise lack population denominators and cannot independently establish drug-induced diabetes [83,84].

A secondary ODYSSEY OUTCOMES biomarker analysis further separates overall treatment safety from associations among metabolic changes. Alirocumab remained globally neutral for incident diabetes, while changes in LDL and Lp(a) showed associations of different directions with that outcome. The biomarker changes were not randomised independently, and the selected subset and exploratory models restrict causal interpretation. These findings neither establish a diabetogenic effect of Lp(a) lowering nor turn overall glycaemic neutrality into evidence of glucose-lowering efficacy [85].

The experimental fusion molecule MEDI4166 combined a PCSK9 antibody with a glucagon-like peptide 1 (GLP-1) analogue. Its phase 1 study showed lipid lowering without a convincing clinically relevant glycaemic effect, in the setting of limited GLP-1 activity and exposure. This does not invalidate incretin biology or demonstrate a glucose benefit from PCSK9 inhibition. It illustrates why the activity of the two components must be assessed separately rather than inferred from the combined molecule [86].

### 9.3. Inclisiran Requires Its Own Evidentiary Assessment

Pooled ORION-9, ORION-10 and ORION-11 analyses document similar substantial LDL lowering across glycaemic categories, with follow-up focused on lipid efficacy and safety. These publications do not establish an immunometabolic cardiovascular benefit. The ORION-1 immune analysis found no consistent reduction of TNF-α or interleukin 6 (IL-6) and no coherent dose-related immune-cell effect. Lack of an inflammatory increase is a safety observation, not demonstration of anti-inflammatory efficacy [17,18].

Direct exposure of macrophages to inclisiran in vitro does not automatically model the distribution and target engagement of a hepatically directed clinical treatment. A cell study without adequate verification of PCSK9 silencing and appropriate sequence controls cannot establish a PCSK9-specific immune class effect. Clinical statements about siRNA should therefore remain grounded in its own treatment evidence [87].

### 9.4. Residual Lipid and Inflammatory Risk

Lp(a), apoB, remnant particles and lipoprotein composition broaden the metabolic consequences of treatment. In ODYSSEY OUTCOMES, changes in Lp(a) were associated with cardiovascular benefit after adjustment for LDL-related change. However, the biomarker changes were not separately randomised, and Lp(a) itself is a lipoprotein exposure. Even a causal contribution from Lp(a) reduction would not by itself constitute a non-lipid mechanism [88].

Triglyceride analyses from the same outcome trial provide a useful counterpoint. Higher baseline triglycerides identified greater risk, but the triglyceride reduction during alirocumab treatment was not associated with fewer subsequent events in the reported model. This does not exclude every contribution from triglyceride-rich lipoproteins, because triglyceride concentration is not identical to remnant cholesterol or particle number. It does show why a baseline risk association cannot by itself establish that changing that biomarker mediates treatment benefit. These secondary reports share ODYSSEY OUTCOMES participants and should not be counted as independent trials [89].

Postprandial and lipidomic studies require similar precision. A small crossover trial in men with type 2 diabetes found lower concentrations of several lipid measures after alirocumab but no significant reduction in the incremental triglyceride response to a meal. Lower total exposure is not the same endpoint as reduced incremental postprandial excursion. Nuclear magnetic resonance (NMR) composition also does not directly measure particle production or clearance [90].

Stable-isotope studies address a different endpoint. In an observational analysis of 148 men, including 28 with type 2 diabetes, circulating PCSK9 was associated with production of apoB-48-containing particles rather than their clearance. The association adds physiological context to postprandial concentrations but does not demonstrate that antibody treatment reduces intestinal production or that such a change mediates cardiovascular events. The assembled kinetic datasets also should not be counted as wholly new independent populations [91].

EXCEED-BHS3 analyses of LDL and HDL characteristics describe related aspects of the same trial population. They should not be counted as independent replications. Their results support lipoprotein remodelling, while subgroup analysis and reporting inconsistencies limit stronger causal interpretations. Table 2 summarises the main clinical evidence and the conclusions it can support [92,93].

Residual lipid risk and residual inflammatory risk can coexist. In the FOURIER inflammatory-risk analysis, evolocumab did not reduce high-sensitivity C-reactive protein, and both inflammatory status and on-treatment LDL-C remained associated with subsequent cardiovascular risk. Relative treatment benefit was consistent across baseline inflammatory strata, whereas higher baseline risk permitted greater absolute benefit. These findings distinguish the prognostic importance of inflammation from evidence that PCSK9 inhibition directly suppresses it [94]. Accordingly, reductions in lipoprotein(a), apolipoprotein B-containing particles or remnants remain lipoprotein effects, even when they influence inflammatory biology; neither biomarker adjustment nor persistent inflammatory risk quantifies a separate non-lipid component of treatment benefit.

## 10. Translational Implications and Research Priorities

The accumulated evidence changes the biological model of PCSK9 more clearly than it changes its present therapeutic classification. PCSK9 influences immune activation, receptor trafficking, platelet responses and organ-specific metabolism. The direction and consequences depend on the cell, substrate, inflammatory stimulus and subcellular compartment. Treating all these findings as one pleiotropic benefit would discard the counterevidence and make the clinical inference less precise. Table 3 summarises what the experimental, translational and randomised evidence can establish.

### 10.1. Contribution Relative to Existing Reviews

The present synthesis builds on established accounts of islet cholesterol homeostasis, glucose metabolism and macrophage activation [8,9,10]. It also agrees with the recent circulatory-inflammation review that plaque remodelling and changes in systemic inflammation are different outcomes [11]. Its contribution is the application of these distinctions to the interpretation of heterogeneous evidence in type 2 diabetes. Table 4 identifies the overlapping scope and the emphasis added here.

The practical consequence is that the apparent contradictions should be examined at the level of the perturbation, compartment and endpoint. The absence of a glycaemic phenotype after β-cell PCSK9 inactivation is relevant counterevidence to models reporting impaired secretion, rather than a reason to assume uniform pancreatic injury [4,13,19,46]. Renal and cardiomyocyte studies likewise show why local PCSK9 loss cannot be assigned the same direction of effect in every tissue [6,7]. Conversely, a direct platelet response in a defined cellular system does not establish how much cardiovascular benefit in patients is mediated by that response [3]. This reading allows genetic diabetes-risk signals and reassuring antibody-trial findings to be interpreted without forcing either to substitute for the other [1,2,76,77].

These distinctions lead to a more specific test of therapeutic claims: the relevant tissue pool must be reached, the mechanism must persist under clinically relevant exposure, and an outcome effect must be separable from accompanying lipoprotein changes. A result can satisfy one of these conditions without satisfying all of them. This is an interpretive framework applied to the assembled evidence, not a validated scoring system or a claim that earlier reviews overlooked these issues.

Established interpretations confirmed here include the compartment distinction, the non-equivalence of genetic deficiency and pharmacological inhibition, and the separation of plaque remodelling from systemic anti-inflammatory efficacy [8,9,10,11]. The synthesis-specific conclusion is the joint interpretation of pancreatic null findings, potentially protective renal and cardiac PCSK9 functions, genetic glycaemic signals and antibody-trial outcomes in type 2 diabetes. Taken together, these observations support an intervention-specific assessment rather than a uniform beneficial or harmful effect of PCSK9 loss. This integrative conclusion does not claim discovery of those individual concepts or an independently validated causal model.

The comparison is selective and intended to clarify scope and interpretation; it is not an exhaustive appraisal or ranking of previous reviews.

### 10.2. Research Priorities and Limitations

Future experiments should define which PCSK9 pool is manipulated and demonstrate target engagement in the relevant cell. Tissue-specific and inducible genetic models can separate developmental effects from adult function. Receptor perturbation, rescue and clinically relevant extracellular exposures can help determine whether a response depends on LDLR, CD36, intracellular cholesterol or another pathway. Comparisons should distinguish measured equivalence of lipid exposure from absence of a statistically significant difference.

Human studies should pair mechanistic measures with designs capable of addressing mediation. Adequately powered randomised imaging or cellular studies, comparators achieving similar apoB and lipoprotein changes, and prespecified longitudinal analyses would be more informative than uncontrolled biomarker correlations. The causal question should be stated before analysis: whether there is a direct cellular effect, whether it changes tissue inflammation, or whether that change contributes to fewer clinical events. These are successive questions rather than one interchangeable endpoint.

For diabetes, renal and pancreatic studies deserve particular attention because local PCSK9 can preserve function in some models. Longer follow-up of glycaemic and organ-specific outcomes remains useful, but experimental knockout injury should not be presented as a clinical adverse effect without treatment evidence. Similarly, favourable cellular findings should not create a renal, hepatic or retinal indication unsupported by human outcomes.

The main limitations of this review are its narrative design, incomplete full-text access and unavailable supplements for some studies. Publication overlap and heterogeneous interventions also prevent simple vote counting. The aim is not to label every finding positive or negative, but to identify which claims are supported at each level of evidence and which require a further causal step.

## 11. Conclusions

PCSK9 participates in immunometabolic processes that extend beyond the regulation of plasma LDL cholesterol. Experimental evidence implicates receptor trafficking, intracellular lipid handling, immune-cell activation, platelet signalling and mitochondrial function, while also showing that PCSK9 loss is not uniformly beneficial across tissues. Local and circulating PCSK9, lifelong genetic deficiency, extracellular antibodies and hepatic silencing must therefore be considered separately. The contribution of this review is to connect these compartment- and intervention-specific findings in a diabetes-focused assessment of which biological observations support clinical claims and which still require direct testing.

In type 2 diabetes, anti-PCSK9 antibodies have established cardiovascular efficacy and an overall reassuring glycaemic profile during the studied follow-up. The available evidence does not yet isolate or quantify a clinically meaningful immunometabolic component of benefit independent of reductions in atherogenic lipoproteins. PCSK9 is consequently a biologically credible immunometabolic target, but current inhibitors should not be described as established glucose-lowering, systemic anti-inflammatory, renal-protective or liver-directed therapies on the basis of mechanistic plausibility alone.

## Figures and Tables

**Figure 1:**
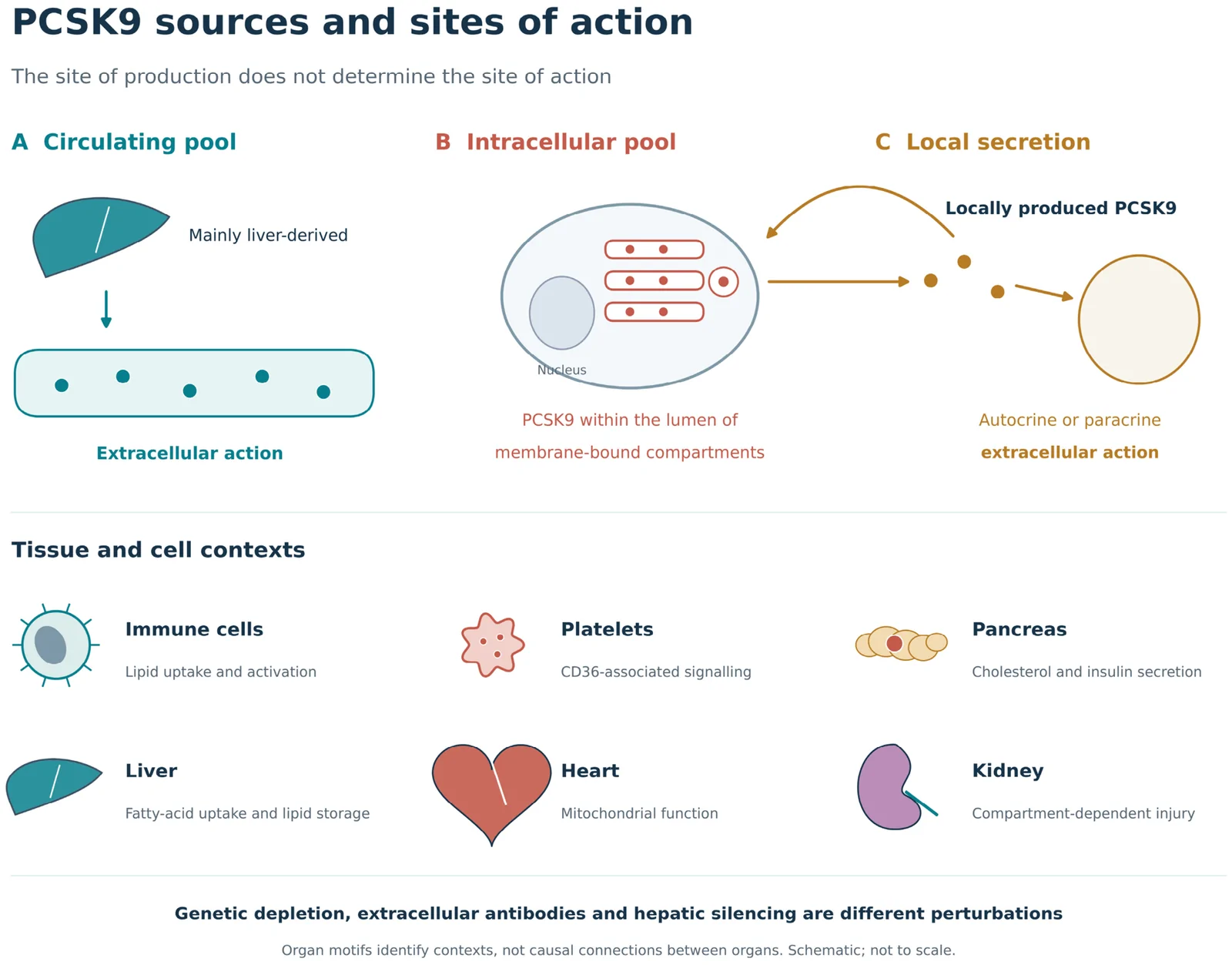
Sources and sites of PCSK9 action in immunometabolic biology. Teal identifies circulating, mainly liver-derived PCSK9 and extracellular exposure; coral identifies intracellular PCSK9 within the lumen of membrane-bound secretory compartments; amber identifies locally secreted PCSK9 with possible autocrine or paracrine extracellular action. Colours distinguish conceptual routes, not chemically different proteins. Local production does not determine the site of action, and locally secreted PCSK9 can also enter the circulation. Arrows in the upper schematic denote secretion or extracellular routes only. The separate organ and cell motifs identify contexts discussed in the review; no arrow connects the heart to the kidney or implies an organ-to-organ causal pathway. Different genetic and pharmacological perturbations are not assumed equivalent. The schematic is not to scale and does not assign the direction of effects in every model. The illustration was prepared programmatically with AI-assisted code; the provenance is documented in Supplementary File S3.

**Table 1:** PCSK9 interventions and the boundaries of mechanistic inference.

Exposure or Intervention	Relevant Compartment	What the Evidence Addresses	Main Limit of Inference
Global knockout	All expressing tissues	Lifelong loss of PCSK9 and combined systemic and local phenotypes [5,13]	Does not reproduce an antibody started in adulthood.
Tissue-specific deletion	Pancreas, heart or other selected tissue	Local receptor trafficking, secretion or mitochondrial function [4,7]	Developmental timing and cell specificity matter.
Recombinant PCSK9 or cellular silencing	Defined experimental cell system	Direct signalling and target-dependent effects [3,15,16]	Dose, target engagement, rescue and biological replication require verification.
Extracellular antibodies	Accessible extracellular PCSK9	Randomised cardiovascular benefit and lipid lowering [1,2]	Concurrent lipoprotein changes prevent automatic attribution to direct immune effects.
Hepatic small interfering RNA (siRNA)	Hepatic PCSK9 production	Lipid efficacy and trial-specific safety findings [17,18]	Not equivalent to systemic or immune-cell gene deletion.
β-cell-specific inactivation	Pancreatic β-cells	Reported preservation of glucose homeostasis and insulin secretion [19]	Abstract only: Peyot et al.; full methods and separate supplements unavailable for verification. Not decisive full-text evidence.

Verification note: main-text findings and experiments delegated to external Supplementary Files are distinguished. For the dendritic-cell silencing study [16], the cited supplementary rescue experiments were not independently available. Supplementary File S2 records the retained appraisal documentation; the present revision did not reacquire every original source full text.

**Table 2:** Selected human evidence and the interpretation supported by study design.

Study or Dataset	Design and Scope	Main Finding	Interpretive Boundary	Verification Limits
FOURIER diabetes analysis [1]	Randomised trial subgroup	Primary cardiovascular endpoint hazard ratio (HR) 0.83 in diabetes; incident diabetes HR 1.05 (95% CI 0.94–1.17).	Cardiovascular efficacy and overall glycaemic reassurance; no separate immune mediation estimate.	Retained main-text appraisal; separate immune mediation not tested.
ODYSSEY OUTCOMES diabetes analysis [2]	Prespecified randomised trial subgroup	Primary endpoint HR 0.84 in diabetes; incident diabetes HR 1.00 (95% CI 0.89–1.11).	Greater absolute benefit in diabetes reflects higher baseline risk, not glucose-lowering efficacy.	Retained main-text appraisal; overlapping trial reports.
PACMAN-AMI PET sub-study [34]	Randomised imaging; 20 analysed	Carotid inflammatory imaging improved; LDL also fell more with alirocumab.	Small surrogate study cannot isolate LDL-independent mediation.	Publisher main text checked; small imaging sub-study.
Seidel and colleagues [38]	Before-and-after; 89 participants	LpPLA2 decreased with a strong LDL relationship; most systemic markers did not improve.	No randomised comparator; no established systemic anti-inflammatory effect.	Retained main-text appraisal; exploratory uncontrolled findings.
ORION-1 immune analysis [18]	Prespecified randomised biomarker analysis	No consistent reduction of TNF-α or IL-6 or coherent dose-related immune effect.	Safety observations do not establish immunometabolic efficacy.	Retained main-text appraisal; unavailable external details not certified.
ORION-9, -10 and -11 [17]	Pooled post hoc subgroup analyses	Substantial LDL reduction across glycaemic categories.	These reports do not establish cardiovascular immune mediation.	Retained main-text appraisal; pooled secondary reports.
DIABHYCAR and SURDIAGENE [67]	Prospective cohorts	PCSK9 prognostic association did not replicate consistently.	No validated universal biomarker threshold or treatment-selection rule.	Retained main-text appraisal; cohort overlap considered.
AMUNDSEN [79]	Randomised early-treatment strategy after acute MI	No significant one-year difference in the main clinical endpoint.	No demonstrated early pleiotropic clinical benefit.	Indexed and publisher abstract checked; external supplement not appraised.
FOURIER inflammatory-risk analysis [94]	Secondary analysis of a randomised trial	Inflammatory risk persisted despite LDL-C lowering.	hsCRP-associated risk does not establish anti-inflammatory mediation.	Indexed abstract checked; external supplement not appraised.

**Table 3:** What each evidence category can establish about PCSK9 immunometabolism.

Evidence Category	Examples and Supported Inference	Main Boundary and Verification Limits
Experimental models	Cellular and receptor perturbation, rescue, global or tissue-specific deletion [3,4,5,6,7,13,15,16]. Supports model-specific mechanisms.	Model, dose, compartment and developmental timing constrain translation. Peyot et al. [19] is abstract-only; unavailable supplemental experiments were not independently verified.
Human genetic evidence	Target-based analyses [76,77,78]. Informs lifelong genetically proxied exposure and possible target-related trade-offs.	Variant, ancestry and instrument assumptions matter; genetically lower LDL-C does not reproduce an adult drug intervention.
Human observational evidence	Circulating PCSK9 cohorts and uncontrolled treatment studies [32,33,36,38,67,68,69,70,71,72]. Identifies associations and candidate pathways.	Confounding, selection, treatment changes and concurrent lipoprotein responses prevent a direct mediation claim.
Randomised mechanistic studies	PACMAN-AMI imaging and ORION-1 biomarkers [18,34]. Tests treatment effects on intermediate endpoints.	Small samples and surrogate outcomes do not establish an independent clinical benefit; missing external details remain unverified.
Randomised antibody outcomes	FOURIER and ODYSSEY OUTCOMES [1,2] establish cardiovascular efficacy. AMUNDSEN [79] adds early-treatment clinical evidence.	Randomisation concerns treatment, not separate lipid and immune mediators. AMUNDSEN access for this revision was limited to indexed and publisher abstracts.
Hepatic silencing	ORION reports [17,18] support LDL-C lowering and trial-specific safety findings.	The reports appraised here do not establish a distinct immunometabolic cardiovascular benefit; hepatic silencing is not immune-cell deletion.

**Table 4:** Relationship to selected existing reviews and the contribution of the present synthesis.

Existing Review	Scope Already Covered	Emphasis of the Present Synthesis
Päth et al., 2022 [8]	β-cell cholesterol handling, knockout models, genetic variants and inhibitor-treated patients; already distinguishes local islet biology from circulating PCSK9.	Extends that compartment distinction to immune and platelet responses and to renal and cardiac counterevidence; tests whether the experimental perturbation models the treatment.
Lu et al., 2023 [9]	Circulating and pancreatic PCSK9, conflicting islet findings, new-onset diabetes and glucose-lowering or natural agents that modify PCSK9.	Keeps changes in PCSK9 during glucose-lowering therapy separate from evidence that PCSK9 inhibition itself lowers glucose; interprets pancreatic findings alongside tissue-specific effects elsewhere.
Katsuki et al., 2024 [10]	Macrophage activation through lipid-dependent, LDLR-dependent and LDLR-independent pathways; acknowledges uncertainty about clinical anti-inflammatory effects.	Applies those mechanistic distinctions to type 2 diabetes across cell and organ systems and asks separately whether a pathway contributes to clinical event reduction.
Circulatory inflammation review, 2026 [11]	Distinguishes direct immunomodulation, lipid-mediated plaque remodelling and systemic biomarkers; includes multisystem findings and translational cautions.	Shares that caution. The present emphasis is the diabetes-specific reconciliation of genetic glycaemic signals, pancreatic counterevidence, organ-specific PCSK9 loss and the reach of antibodies versus hepatic silencing.

## Data Availability

No new data were created or analyzed in this study. Data sharing is not applicable to this article.

## Supplementary Materials

The following supporting information can be downloaded at: https://www.mdpi.com/article/10.3390/ijms27219723/s1.

## Abbreviations

Trial and cohort names are used as study identifiers; gene symbols follow the terminology of the cited publications.

apoB	apolipoprotein B
CD36	cluster of differentiation 36
cGAS	cyclic GMP–AMP synthase
ChREBP	carbohydrate-responsive element-binding protein
CI	confidence interval
CT	computed tomography
DNA	deoxyribonucleic acid
eGFR	estimated glomerular filtration rate
FDG-PET	fluorodeoxyglucose positron emission tomography
GLP-1	glucagon-like peptide 1
HbA1c	glycated haemoglobin
HDL	high-density lipoprotein
HIV	human immunodeficiency virus
HMGCR	3-hydroxy-3-methylglutaryl-coenzyme A reductase
HR	hazard ratio
hsCRP	high-sensitivity C-reactive protein
IL-6	interleukin 6
LDL	low-density lipoprotein
LDL-C	low-density lipoprotein cholesterol
LDLR	low-density lipoprotein receptor
LOX-1	lectin-like oxidised low-density lipoprotein receptor 1
Lp(a)	lipoprotein(a)
LpPLA2	lipoprotein-associated phospholipase A2
mRNA	messenger RNA
NF-κB	nuclear factor κB
NLRP3	NOD-like receptor family pyrin domain containing 3
NMR	nuclear magnetic resonance
OR	odds ratio
PCSK9	proprotein convertase subtilisin/kexin type 9
siRNA	small interfering RNA
SR-A	scavenger receptor class A
STEMI	ST-elevation myocardial infarction
STING	stimulator of interferon genes
TLR4	Toll-like receptor 4
TNF-α	tumour necrosis factor α
VLDL	very-low-density lipoprotein
VLDLR	very-low-density lipoprotein receptor
YAP	yes-associated protein
