# Supplementary material for: PCSK9 in the Immunometabolism of Type 2 Diabetes
Source: Int J Mol Sci. 2026 Oct 29;27(21):9723. doi: 10.3390/ijms27219723 (PMC13763235; doi:10.3390/ijms27219723)
Supplement: Supplementary file 1 [file ijms-27-09723-s001.zip › PCSK9_Supplementary_S1_Search_Strategies(2).pdf]

Supplementary File S1

Literature search strategies for the PCSK9 review

PCSK9 in the Immunometabolism of Type 2 Diabetes

This supplement presents the archived database-specific search strategies for the critical narrative review. It describes the search formulations and their thematic scope; it does not constitute a reproducible systematic-review selection log or establish exhaustive coverage.

The archived PubMed queries specify a publication-date upper limit of 4 October 2026. This limit is part of the query syntax and is not evidence of the date on which a search was executed. For the other platforms, the archived accompanying instructions specify coverage through 2026, with publications after 4 October 2026 excluded during record checking; the actual interface settings cannot be independently verified from this document.

Execution dates, per-query retrieval counts, final interface modifications and Web of Science citation-index coverage were not recorded in the supplied search document. They are therefore not reported here. The appraisal corpus described in the manuscript is distinct from the initial search yields.

PubMed

The strategies use title and abstract terms, with selected MeSH and publication-type terms. Q0 is a broad search; Q1 to Q6 cover the main themes. Q7 targets recent reviews from 2024 to 4 October 2026. The archived search plan specifies no initial language, species, free-full-text or study-design filters for Q0 to Q6.

Q0 Broad PCSK9 search

(PCSK9[tiab] OR "PCSK-9"[tiab] OR "proprotein convertase subtilisin/kexin type 9"[tiab] OR "proprotein convertase subtilisin kexin type 9"[tiab]) AND ("1900/01/01"[dp] : "2026/10/04"[dp])

Q1 Diabetes and metabolic biology

(PCSK9[tiab] OR "PCSK-9"[tiab] OR "proprotein convertase subtilisin/kexin type 9"[tiab] OR "proprotein convertase subtilisin kexin type 9"[tiab]) AND ("Diabetes Mellitus, Type 2"[Mesh] OR diabet*[tiab] OR "insulin resistance"[tiab] OR hyperglyc*[tiab] OR hyperinsulin*[tiab] OR obes*[tiab] OR adipos*[tiab] OR "beta cell*"[tiab] OR "beta-cell*"[tiab] OR "insulin secretion"[tiab]) AND ("1900/01/01"[dp] : "2026/10/04"[dp])

Q2 Immunometabolism and endothelium

(PCSK9[tiab] OR "PCSK-9"[tiab] OR "proprotein convertase subtilisin/kexin type 9"[tiab] OR "proprotein convertase subtilisin kexin type 9"[tiab]) AND (inflamm*[tiab] OR immunometabol*[tiab] OR macrophag*[tiab] OR "foam cell*"[tiab] OR NLRP3[tiab] OR TLR4[tiab] OR "NF-kappa B"[tiab] OR "NF-kB"[tiab] OR "NF-κB"[tiab] OR "oxidative stress"[tiab] OR endotheli*[tiab] OR "LOX-1"[tiab] OR CD36[tiab]) AND ("1900/01/01"[dp] : "2026/10/04"[dp])

Q3 Molecular regulation and compartments

(PCSK9[tiab] OR "PCSK-9"[tiab] OR "proprotein convertase subtilisin/kexin type 9"[tiab] OR "proprotein convertase subtilisin kexin type 9"[tiab]) AND (LDLR[tiab] OR "LDL receptor*"[tiab] OR SREBP*[tiab] OR HNF1*[tiab] OR insulin[tiab] OR intracell*[tiab] OR secret*[tiab] OR hepat*[tiab] OR liver[tiab] OR adipos*[tiab] OR renal[tiab] OR kidney[tiab]) AND ("1900/01/01"[dp] : "2026/10/04"[dp])

Q4 Treatments and glycaemic safety

(alirocumab[tiab] OR evolocumab[tiab] OR inclisiran[tiab] OR "PCSK9 inhibitor*"[tiab] OR "PCSK9 inhibition"[tiab] OR "PCSK9-target*"[tiab]) AND (diabet*[tiab] OR glycaem*[tiab] OR glycem*[tiab] OR HbA1c[tiab] OR glucose[tiab] OR "insulin resistance"[tiab]) AND ("1900/01/01"[dp] : "2026/10/04"[dp])

Q5 Residual risk biomarkers and imaging

((PCSK9[tiab] OR "PCSK-9"[tiab] OR "proprotein convertase subtilisin/kexin type 9"[tiab] OR "proprotein convertase subtilisin kexin type 9"[tiab]) OR (alirocumab[tiab] OR evolocumab[tiab] OR inclisiran[tiab] OR "PCSK9 inhibitor*"[tiab] OR "PCSK9 inhibition"[tiab] OR "PCSK9-target*"[tiab])) AND ("residual risk"[tiab] OR apoB[tiab] OR apolipoprotein*[tiab] OR remnant*[tiab] OR "lipoprotein(a)"[tiab] OR "Lp(a)"[tiab] OR hsCRP[tiab] OR "C-reactive protein"[tiab] OR cytokine*[tiab] OR inflamm*[tiab] OR endotheli*[tiab] OR plaque[tiab] OR imaging[tiab]) AND ("1900/01/01"[dp] : "2026/10/04"[dp])

Q6 Genetics and loss of function

(PCSK9[tiab] OR "PCSK-9"[tiab] OR "proprotein convertase subtilisin/kexin type 9"[tiab] OR "proprotein convertase subtilisin kexin type 9"[tiab]) AND ("Mendelian randomization"[tiab] OR "Mendelian randomisation"[tiab] OR "loss of function"[tiab] OR "loss-of-function"[tiab] OR knockout[tiab] OR variant*[tiab]) AND (diabet*[tiab] OR glucose[tiab] OR insulin[tiab]) AND ("1900/01/01"[dp] : "2026/10/04"[dp])

Q7 Recent reviews and related syntheses

((PCSK9[tiab] OR "PCSK-9"[tiab] OR "proprotein convertase subtilisin/kexin type 9"[tiab] OR "proprotein convertase subtilisin kexin type 9"[tiab]) OR (alirocumab[tiab] OR evolocumab[tiab] OR inclisiran[tiab] OR "PCSK9 inhibitor*"[tiab] OR "PCSK9 inhibition"[tiab] OR "PCSK9-target*"[tiab])) AND (diabet*[tiab] OR immunometabol*[tiab] OR inflamm*[tiab] OR "residual risk"[tiab]) AND (review[pt] OR "systematic review"[pt] OR review[ti]) AND ("2024/01/01"[dp] : "2026/10/04"[dp])

Embase.com

The strategies use free-text terms in titles, abstracts and author keywords with the :ti,ab,kw field code. These formulations apply to Embase.com, not Ovid. No Emtree terms are included. The archived plan specifies all available years through 2026 for Q0 to Q6 and 2024 to 2026 for Q7, without language or full-text-availability restrictions.

Q0 Broad PCSK9 search

((PCSK9 OR 'PCSK-9' OR 'proprotein convertase subtilisin/kexin type 9' OR 'proprotein convertase subtilisin kexin type 9')):ti,ab,kw

Q1 Diabetes and metabolic biology

((PCSK9 OR 'PCSK-9' OR 'proprotein convertase subtilisin/kexin type 9' OR 'proprotein convertase subtilisin kexin type 9') AND (diabet* OR 'insulin resistance' OR hyperglyc* OR hyperinsulin* OR obes* OR adipos* OR 'beta cell*' OR 'beta-cell*' OR 'insulin secretion')):ti,ab,kw

Q2 Immunometabolism and endothelium

((PCSK9 OR 'PCSK-9' OR 'proprotein convertase subtilisin/kexin type 9' OR 'proprotein convertase subtilisin kexin type 9') AND (inflamm* OR immunometabol* OR macrophag* OR 'foam cell*' OR NLRP3 OR TLR4 OR 'NF-kappa B' OR 'NF-kB' OR 'NF-κB' OR 'oxidative stress' OR endotheli* OR 'LOX-1' OR CD36)):ti,ab,kw

Q3 Molecular regulation and compartments

((PCSK9 OR 'PCSK-9' OR 'proprotein convertase subtilisin/kexin type 9' OR 'proprotein convertase subtilisin kexin type 9') AND (LDLR OR 'LDL receptor*' OR SREBP* OR HNF1* OR insulin OR intracell* OR secret* OR hepat* OR liver OR adipos* OR renal OR kidney)):ti,ab,kw

Q4 Treatments and glycaemic safety

((alirocumab OR evolocumab OR inclisiran OR 'PCSK9 inhibitor*' OR 'PCSK9 inhibition' OR 'PCSK9-target*') AND (diabet* OR glycaem* OR glycem* OR HbA1c OR glucose OR 'insulin resistance')):ti,ab,kw

Q5 Residual risk biomarkers and imaging

(((PCSK9 OR 'PCSK-9' OR 'proprotein convertase subtilisin/kexin type 9' OR 'proprotein convertase subtilisin kexin type 9') OR (alirocumab OR evolocumab OR inclisiran OR 'PCSK9 inhibitor*' OR 'PCSK9 inhibition' OR 'PCSK9-target*')) AND ('residual risk' OR apoB OR apolipoprotein* OR remnant* OR 'lipoprotein(a)' OR 'Lp(a)' OR hsCRP OR 'C-reactive protein' OR cytokine* OR inflamm* OR endotheli* OR plaque OR imaging)):ti,ab,kw

Q6 Genetics and loss of function

((PCSK9 OR 'PCSK-9' OR 'proprotein convertase subtilisin/kexin type 9' OR 'proprotein convertase subtilisin kexin type 9') AND ('Mendelian randomization' OR 'Mendelian randomisation' OR 'loss of function' OR 'loss-of-function' OR knockout OR variant*) AND (diabet* OR glucose OR insulin)):ti,ab,kw

Q7 Recent reviews and related syntheses

(((PCSK9 OR 'PCSK-9' OR 'proprotein convertase subtilisin/kexin type 9' OR 'proprotein convertase subtilisin kexin type 9') OR (alirocumab OR evolocumab OR inclisiran OR 'PCSK9 inhibitor*' OR 'PCSK9 inhibition' OR 'PCSK9-target*')) AND (diabet* OR immunometabol* OR inflamm* OR 'residual risk') AND (review OR 'meta-analysis' OR 'meta analysis')):ti,ab,kw

Web of Science Core Collection

The TS field covers titles, abstracts, author keywords and Keywords Plus. The archived plan specifies all available years through 2026 for Q0 to Q6 and 2024 to 2026 for Q7, without language or full-text-availability restrictions. The specific citation indexes accessed are not documented.

Q0 Broad PCSK9 search

TS=((PCSK9 OR "PCSK-9" OR "proprotein convertase subtilisin/kexin type 9" OR "proprotein convertase subtilisin kexin type 9"))

Q1 Diabetes and metabolic biology

TS=((PCSK9 OR "PCSK-9" OR "proprotein convertase subtilisin/kexin type 9" OR "proprotein convertase subtilisin kexin type 9") AND (diabet* OR "insulin resistance" OR hyperglyc* OR hyperinsulin* OR obes* OR adipos* OR "beta cell*" OR "beta-cell*" OR "insulin secretion"))

Q2 Immunometabolism and endothelium

TS=((PCSK9 OR "PCSK-9" OR "proprotein convertase subtilisin/kexin type 9" OR "proprotein convertase subtilisin kexin type 9") AND (inflamm* OR immunometabol* OR macrophag* OR "foam cell*" OR NLRP3 OR TLR4 OR "NF-kappa B" OR "NF-kB" OR "NF-κB" OR "oxidative stress" OR endotheli* OR "LOX-1" OR CD36))

Q3 Molecular regulation and compartments

TS=((PCSK9 OR "PCSK-9" OR "proprotein convertase subtilisin/kexin type 9" OR "proprotein convertase subtilisin kexin type 9") AND (LDLR OR "LDL receptor*" OR SREBP* OR HNF1* OR insulin OR intracell* OR secret* OR hepat* OR liver OR adipos* OR renal OR kidney))

Q4 Treatments and glycaemic safety

TS=((alirocumab OR evolocumab OR inclisiran OR "PCSK9 inhibitor*" OR "PCSK9 inhibition" OR "PCSK9-target*") AND (diabet* OR glycaem* OR glycem* OR HbA1c OR glucose OR "insulin resistance"))

Q5 Residual risk biomarkers and imaging

TS=(((PCSK9 OR "PCSK-9" OR "proprotein convertase subtilisin/kexin type 9" OR "proprotein convertase subtilisin kexin type 9") OR (alirocumab OR evolocumab OR inclisiran OR "PCSK9 inhibitor*" OR "PCSK9 inhibition" OR "PCSK9-target*")) AND ("residual risk" OR apoB OR apolipoprotein* OR remnant* OR "lipoprotein(a)" OR "Lp(a)" OR hsCRP OR "C-reactive protein" OR cytokine* OR inflamm* OR endotheli* OR plaque OR imaging))

Q6 Genetics and loss of function

TS=((PCSK9 OR "PCSK-9" OR "proprotein convertase subtilisin/kexin type 9" OR "proprotein convertase subtilisin kexin type 9") AND ("Mendelian randomization" OR "Mendelian randomisation" OR "loss of function" OR "loss-of-function" OR knockout OR variant*) AND (diabet* OR glucose OR insulin))

Q7 Recent reviews and related syntheses

TS=(((PCSK9 OR "PCSK-9" OR "proprotein convertase subtilisin/kexin type 9" OR "proprotein convertase subtilisin kexin type 9") OR (alirocumab OR evolocumab OR inclisiran OR "PCSK9 inhibitor*" OR "PCSK9 inhibition" OR "PCSK9-target*")) AND (diabet* OR immunometabol* OR inflamm* OR "residual risk") AND (review OR "meta-analysis" OR "meta analysis"))

CENTRAL

The archived strategy is intended for the Trials collection in the Cochrane Library Search Manager. Line 4 reproduces the broad set from line 1; lines 5 and 6 identify metabolic and biological or imaging subsets. These overlapping subsets must not be summed as independent search yields. No additional randomised-trial filter is included.

Line 1 Target and treatments

(PCSK9 OR "PCSK-9" OR "proprotein convertase subtilisin/kexin type 9" OR "proprotein convertase subtilisin kexin type 9" OR alirocumab OR evolocumab OR inclisiran OR "PCSK9 inhibitor" OR "PCSK9 inhibitors"):ti,ab,kw

Line 2 Diabetes and glycaemia

(diabet* OR glycaem* OR glycem* OR glucose OR HbA1c OR insulin):ti,ab,kw

Line 3 Biomarkers and vascular wall

(inflamm* OR hsCRP OR "C-reactive protein" OR cytokine* OR endotheli* OR plaque OR imaging OR "lipoprotein(a)" OR "Lp(a)"):ti,ab,kw

Line 4 Broad result set

#1

Line 5 Metabolic subset

#1 AND #2

Line 6 Biological and imaging subset

#1 AND #3

Syntax documentation

https://pubmed.ncbi.nlm.nih.gov/help/

https://www.elsevier.support/embase/answer/what-field-codes-can-i-use-in-embase

https://www.elsevier.support/embase/answer/how-do-i-search-in-embase

https://webofscience.help.clarivate.com/en-us/Content/wos-core-collection/woscc-search-field-tags.htm

https://resources.cochrane.org/sites/resources.cochrane.org/files/uploads/Trainers/Activity%202_Cochrane%20Library_preview.pdf

Targeted additions during peer review

On 23 October 2026, targeted title and DOI lookups addressed the reviewer-requested AMUNDSEN trial (10.1001/jama.2026.17302) and the FOURIER inflammatory-risk analysis (10.1161/CIRCULATIONAHA.118.034032). Indexed publication records and accessible publisher material were checked. This was targeted retrieval, not a rerun of the archived four-database searches. No new database search yield, execution date for the original searches or systematic-review selection count is inferred from this step. The original search strings above are unchanged. Verification levels for the two additions are recorded in Supplementary File S2.
