# Supplementary material for: PCSK9 in the Immunometabolism of Type 2 Diabetes
Source: Int J Mol Sci. 2026 Oct 29;27(21):9723. doi: 10.3390/ijms27219723 (PMC13763235; doi:10.3390/ijms27219723)
Supplement: Supplementary file 1 [file ijms-27-09723-s001.zip › PCSK9_Supplementary_S2_Evidence_Inventory_2.pdf]

PCSK9 evidence inventory

Supplementary File S2
Reconciliation of the original 108 appraised publications and 94 revised manuscript references

Manuscript ijms-4662068
PCSK9 in the Immunometabolism of Type 2 Diabetes
Reference numbering follows the peer-review revision prepared on 23 October 2026. The submitted version dated 15 October 2026 contained 68 references; the editorial revision dated 17 October contained 92.

Purpose and verified counts

This inventory links the publications documented in the retained critical appraisal records to the bibliography of the revised manuscript. The review is a critical narrative review informed by structured searches. This inventory is not a systematic-review eligibility log or a PRISMA study flow.

- 108 distinct publications have identifiable individual appraisal records: 98 from the original 113-record full-text retrieval list and 10 additional publications.
- 85 of the original 108 appraised publications are cited in the revised manuscript: 77 from the retrieval list and eight additional publications. The submitted version cited 68; 17 were integrated during editorial revision. Three targeted evidence additions and four contextual reviews brought the bibliography to 92 before external peer review. Two further peer-review additions, AMUNDSEN and the FOURIER inflammatory-risk analysis, bring the current bibliography to 94. All nine additions outside the historical corpus are documented separately. The original corpus remains 85 cited plus 23 inventory-only publications.
- 23 appraised publications remain without individual citation: 21 from the retrieval list and two additional publications. The revision-stage reassessment of all 40 initially uncited publications is documented below.
- 15 of the 113 retrieval records are recorded as unavailable in full text in the final synthesis. They are listed separately and are not included in the 108 appraised publications.

Overall strengths and limitations by study category

This is a qualitative interpretation of study design and verification limits. It is not a formal GRADE assessment, a numerical risk-of-bias score or a count of independent cohorts. The categories overlap and are not intended for vote counting.

Evidence category	Main strength	Main limitation
Experimental cell and animal models	Perturbation, receptor dependence and rescue can identify a mechanism within a specified model.	Dose, developmental timing, species and compartment limit drug translation. Conflicting organ phenotypes are retained. Abstract-only or missing-supplement findings remain explicitly qualified.
Human genetic studies	Lifelong target-related variation provides complementary evidence about cardiovascular and glycaemic trade-offs.	Instrument assumptions, ancestry, exposure scaling, pleiotropy and population overlap prevent direct estimation of an adult drug effect.
Human observational and biomarker studies	Provides clinically relevant associations, temporal observations and candidate mediators.	Confounding, selection, treatment changes and inconsistent replication prevent causal mediation claims. LDL adjustment is not proof of lipid independence.

Evidence category	Main strength	Main limitation
Mechanistic treatment and imaging studies	Randomised designs can establish an effect on a prespecified intermediate outcome.	Small samples, surrogates and simultaneous lipoprotein changes limit inference about clinical benefit; uncontrolled studies add further uncertainty.
Randomised clinical outcomes	Provides the strongest treatment-effect evidence for the population, intervention and follow-up studied.	Subgroups, secondary reports and shared participants are not independent replications. Treatment randomisation does not separately randomise lipid and immune mediators.
Hepatic silencing studies	Direct evidence for the lipid efficacy and safety endpoints measured in the appraised reports.	Does not reproduce systemic or immune-cell gene deletion, and the reports considered here do not establish an additional immunometabolic clinical benefit.

Interpretation of citation status

Appraisal and citation are different stages of the narrative synthesis. An appraisal record documents the assessment of a publication; a manuscript reference identifies a publication explicitly cited in the revised text. The 23 publications without a manuscript reference remain part of the documented appraisal corpus. Their absence from the bibliography is not a systematic-review eligibility exclusion.

During editorial revision, the 40 initially uncited publications were reassessed individually against the scope and claims of the manuscript. Seventeen were integrated to add relevant mechanisms, human findings or counterevidence; 23 remained in the inventory because of limited incremental contribution or peripheral scope. The decisions below were made during revision; they are not presented as contemporaneous explanations for the original omissions. This was a reassessment of retained appraisal records, not a fresh acquisition or rereading of all source full texts.

Source records and matching

Source A is PCSK9_Valutazione_critica_riconciliata.docx, version 2 dated 7 October 2026, recovered from the retained review documentation. It contains 64 individually numbered appraisal records: 55 retrieval-list publications and nine additional publications. Source B is PCSK9_Sintesi_critica_e_conclusione(1).docx, containing 44 further appraisal records: 43 retrieval-list publications and one additional publication by Amput and colleagues. Together these identify 108 distinct publications by DOI, without overlap between the two appraisal blocks.

The original 41-PDF appraisal document is an earlier subset of Source A and is not counted again. PCSK9_registro_recupero_fulltext(1).xlsx supplies original retrieval numbers, record identifiers and bibliographic metadata. Its access statuses describe an earlier recovery stage; later individual appraisal records supersede those statuses where a main text was subsequently obtained.

Matching to manuscript references used DOI, ignoring letter case and terminal punctuation. For retrieval record 84 the corrected DOI is 10.4149/gpb_2019057. 85 revised references match the original appraisal corpus. The three targeted additions are listed separately with their verification level, rather than being retrospectively counted in the original 108-publication corpus. New bibliography entries were checked against Europe PMC bibliographic metadata on 17 October 2026. Publications, rather than independent cohorts, are counted: secondary analyses and pooled reports may share trial populations. This reconciliation checks documentary traceability and does not constitute a new full-text appraisal or independent confirmation of all recorded findings.

How to read the inventory

R denotes the original retrieval-list number and its stable record ID. E01–E10 are additional-publication identifiers assigned for this inventory. A numbers refer to the numbered appraisal record in Source A. B numbers refer to the original article heading in the 44-record block of Source B; B extra is its additional Amput record. Reference numbers below refer exclusively to the revised 94-reference bibliography. A dash means no individual citation in that version.

Original corpus publications cited in the manuscript

85 publications. Numbering follows the revised bibliography.

Inventory ID	Publication and DOI	Appraisal record	Manuscript reference
R011<br>P06622	Cardiovascular safety and efficacy of the PCSK9 inhibitor evolocumab in patients with and without diabetes and the effect of evolocumab on glycaemia and risk of new-onset diabetes: a prespecified analysis of the FOURIER randomised controlled trial.<br>DOI 10.1016/s2213-8587(17)30313-3	A50	1
R025<br>P06763	Effects of alirocumab on cardiovascular and metabolic outcomes after acute coronary syndrome in patients with or without diabetes: a prespecified analysis of the ODYSSEY OUTCOMES randomised controlled trial.<br>DOI 10.1016/s2213-8587(19)30158-5	A52	2
R063<br>P00009	PCSK9 (Proprotein Convertase Subtilisin/Kexin 9) Enhances Platelet Activation, Thrombosis, and Myocardial Infarct Expansion by Binding to Platelet CD36.<br>DOI 10.1161/circulationaha.120.046290	B63	3
R062<br>P03783	Pancreatic PCSK9 controls the organization of the β-cell secretory pathway via LDLR-cholesterol axis.<br>DOI 10.1016/j.metabol.2022.155291	B62	4
R075<br>P00277	PCSK9 Induces CD36 Degradation and Affects Long-Chain Fatty Acid Uptake and Triglyceride Metabolism in Adipocytes and in Mouse Liver.<br>DOI 10.1161/atvbaha.115.306032	A25	5
R110<br>P04098	The role of PCSK9 in glomerular lipid accumulation and renal injury in diabetic kidney disease.<br>DOI 10.1007/s00125-024-06191-8	A55	6
R010<br>P05027	Cardiomyocyte-specific PCSK9 deficiency compromises mitochondrial bioenergetics and heart function.<br>DOI 10.1093/cvr/cvad041	A5	7
R015<br>P04175	Circulating proprotein convertase subtilisin/kexin 9 (PCSK9) regulates VLDLR protein and triglyceride accumulation in visceral adipose tissue.<br>DOI 10.1161/atvbaha.110.220988	B15	12
R068<br>P03853	PCSK9 deficiency reduces insulin secretion and promotes glucose intolerance: the role of the low-density lipoprotein receptor.<br>DOI 10.1093/eurheartj/ehy357	A54	13
R086<br>P00130	PCSK9 Regulates Nox2-Mediated Platelet Activation via CD36 Receptor in Patients with Atrial Fibrillation.<br>DOI 10.3390/antiox9040296	A30	14
R074<br>P00836	PCSK9 induces a pro-inflammatory response in macrophages.<br>DOI 10.1038/s41598-018-20425-x	A24	15

Inventory ID	Publication and DOI	Appraisal record	Manuscript reference
R081<br>P00524	PCSK9 plays a novel immunological role in oxidized LDL-induced dendritic cell maturation and activation of T cells from human blood and atherosclerotic plaque.<br>DOI 10.1111/joim.12758	B81	16
R043<br>P07538	Inclisiran in individuals with diabetes or obesity: Post hoc pooled analyses of the ORION-9, ORION-10 and ORION-11 Phase 3 randomized trials.<br>DOI 10.1111/dom.15650	A53	17
R022<br>P00550	Effect of inclisiran, the small-interfering RNA against proprotein convertase subtilisin/kexin type 9, on platelets, immune cells, and immunological biomarkers: a pre-specified analysis from ORION-1.<br>DOI 10.1093/cvr/cvaa077	A51	18
R085<br>P00086	PCSK9 regulates expression of scavenger receptors and ox-LDL uptake in macrophages.<br>DOI 10.1093/cvr/cvy079	B85	20
R059<br>P00222	New role of PCSK9 in atherosclerotic inflammation promotion involving the TLR4/NF-κB pathway.<br>DOI 10.1016/j.atherosclerosis.2017.04.023	B59	21
R093<br>P00230	Proprotein convertase subtilisin kexin type 9 (PCSK9) secreted by cultured smooth muscle cells reduces macrophages LDLR levels.<br>DOI 10.1016/j.atherosclerosis.2011.11.026	B93	22
R012<br>P00262	Circulating levels of proprotein convertase subtilisin/kexin type 9 (PCSK9) are associated with monocyte subsets in patients with stable coronary artery disease.<br>DOI 10.1016/j.jacl.2021.02.005	B12	23
E02<br>P00043	NLRP3 inflammasome via IL-1β regulates PCSK9 secretion.<br>DOI 10.7150/thno.45939	A57	24
R094<br>P00224	Proprotein Convertase Subtilisin/Kexin Type 9 (PCSK9) Is Not Induced in Artificial Human Inflammation and Is Not Correlated with Inflammatory Response.<br>DOI 10.1128/iai.00842-19	A33	25
R008<br>P00059	Blood flow patterns regulate PCSK9 secretion via MyD88-mediated pro-inflammatory cytokines.<br>DOI 10.1093/cvr/cvz262	A4	26
R072<br>P00438	PCSK9 Expression in Vascular Smooth Muscle Cells: Role of Insulin Resistance and High Glucose.<br>DOI 10.3390/ijms26031003	A23	27
R066<br>P00187	PCSK9 Confers Inflammatory Properties to Extracellular Vesicles Released by Vascular Smooth Muscle Cells.<br>DOI 10.3390/ijms232113065	A20	28
R056<br>P00231	Modulation of vascular endothelial inflammatory response by proprotein convertase subtilisin-kexin type 9.<br>DOI 10.1016/j.atherosclerosis.2022.09.008	B56	29
R083<br>P00050	PCSK9 Promotes Hypoxia-Induced EC Pyroptosis by Regulating Smac Mitochondrion-Cytoplasm Translocation in Critical Limb Ischemia.<br>DOI 10.1016/j.jacbts.2023.05.016	A29	30

Inventory ID	Publication and DOI	Appraisal record	Manuscript reference
R089 P00357	PCSK9/LOX-1 is associated with T2DM and regulates high glucose-induced lipid metabolism dysfunction in human microvascular endothelial cells. DOI 10.5603/ep.101052	A31	31
E07 P00518	Association of PCSK9 with inflammation and platelet activation markers and recurrent cardiovascular risks in STEMI patients undergoing primary PCI with or without diabetes. DOI 10.1186/s12933-022-01519-3	A62	32
R023 P07465	Effect of proprotein convertase subtilisin kexin 9 inhibitors on platelet aggregation in patients with and without diabetes. DOI 10.1016/j.numecd.2026.104728	B23	33
R019 P03232	Effect of Alirocumab on Carotid Inflammation by [(18)F]FDG PET in Patients With Acute Myocardial Infarction. DOI 10.1016/j.jcmg.2024.04.001	B19	34
R041 P01945	Impact of Diabetes on Coronary Plaque Stabilization With Evolocumab: Insights From the YELLOW III Trial. DOI 10.1007/s10557-026-07962-8	B41	35
E05 P02154	Evolocumab attenuate pericoronary adipose tissue density via reduction of lipoprotein(a) in type 2 diabetes mellitus: a serial follow-up CCTA study. DOI 10.1186/s12933-023-01857-w	A60	36
R032 P00189	Evolocumab, a PCSK9-Monoclonal Antibody, Rapidly Reverses Coronary Artery Endothelial Dysfunction in People Living With HIV and People With Dyslipidemia. DOI 10.1161/jaha.120.016263	A12	37
R007 P00560	Beyond lipid lowering: Effects of PCSK9 inhibition on inflammation and HDL function. DOI 10.1016/j.jacl.2026.02.003	B7	38
R001 P01103	[Comparison of the efficacy of PCSK9 inhibitors and statins on dyslipidemia in patients with type 2 diabetes]. DOI 10.3760/cma.j.cn112137-20220325-00630	B1	39
R034 P00068	Gene Expression Profiling of Markers of Inflammation, Angiogenesis, Coagulation and Fibrinolysis in Patients with Coronary Artery Disease with Very High Lipoprotein(a) Levels Treated with PCSK9 Inhibitors. DOI 10.3390/jcdd9070211	A13	40
R097 P00670	Residual Inflammatory Risk on Treatment With PCSK9 Inhibition and Statin Therapy. DOI 10.1161/circulationaha.118.034645	A48	41
R049 P00759	Intensive Lowering of LDL Cholesterol Levels With Evolocumab in Autoimmune or Inflammatory Diseases: An Analysis of the FOURIER Trial. DOI 10.1161/circulationaha.124.072756	A16	42
R078 P03812	PCSK9 is expressed in pancreatic delta-cells and does not alter insulin secretion. DOI 10.1016/j.bbrc.2009.10.138	B78	43
R088 P00065	PCSK9-deficient mice exhibit impaired glucose tolerance and pancreatic islet abnormalities. DOI 10.1016/j.febslet.2009.12.018	B88	44

Inventory ID	Publication and DOI	Appraisal record	Manuscript reference
R113<br>P04209	Variable effects of gender and Western diet on lipid and glucose homeostasis in aged PCSK9-deficient C57BL/6 mice.<br>DOI 10.1111/1753-0407.12139	B113	45
R027<br>P03767	Effects of proprotein convertase subtilisin kexin type 9 modulation in human pancreatic beta cells function.<br>DOI 10.1016/j.atherosclerosis.2021.03.044	A42	46
R035<br>P02661	Hepatic PCSK9 expression is regulated by nutritional status via insulin and sterol regulatory element-binding protein 1c.<br>DOI 10.1074/jbc.m508582200	B35	47
R099<br>P03781	The Role of Insulin in the Regulation of PCSK9<br>DOI 10.1161/atvbaha.115.305688	A49	48
R106<br>P03728	The effect of insulin on circulating PCSK9 in postmenopausal obese women.<br>DOI 10.1016/j.clinbiochem.2014.03.022	B106	49
R058<br>P04063	New Insight Into Metformin-Induced Cholesterol-Lowering Effect Crosstalk Between Glucose and Cholesterol Homeostasis via ChREBP (Carbohydrate-Responsive Element-Binding Protein)-Mediated PCSK9 (Proprotein Convertase Subtilisin/Kexin Type 9) Regulation.<br>DOI 10.1161/atvbaha.120.315708	B58	50
R102<br>P04158	Statin-Induced Geranylgeranyl Pyrophosphate Depletion Promotes PCSK9-Dependent Adipose Insulin Resistance.<br>DOI 10.3390/nu14245314	A38	51
R065<br>P00002	PCSK9 causes inflammation and cGAS/STING pathway activation in diabetic nephropathy.<br>DOI 10.1096/fj.202300342rrr	B65	52
R052<br>P00422	Lack of renoprotective effects by long-term PCSK9 and SGLT2 inhibition using alirocumab and empagliflozin in obese ZSF1 rats.<br>DOI 10.1152/ajprenal.00065.2024	B52	53
R036<br>P01036	Hepatocyte-specific yes-associated protein knockout exacerbates non-alcoholic steatohepatitis by upregulating PCSK9.<br>DOI 10.1007/s00535-026-02372-x	B36	54
R048<br>P00848	Inhibition of hepatic PCSK9 as a novel therapeutic target ameliorates metabolic steatohepatitis in mice.<br>DOI 10.1016/j.intimp.2024.113621	B48	55
E01<br>P00025	Pcsk9 Deletion Promotes Murine Nonalcoholic Steatohepatitis and Hepatic Carcinogenesis: Role of Cholesterol.<br>DOI 10.1002/hep4.1858	A56	56
R111<br>P04845	The role of PCSK9 in metabolic dysfunction-associated steatotic liver disease and its impact on bariatric surgery outcomes.<br>DOI 10.1016/j.soard.2024.01.017	B111	57
R071<br>P00159	PCSK9 Enhances Cardiac Fibrogenesis via the Activation of Toll-like Receptor and NLRP3 Inflammasome Signaling.<br>DOI 10.3390/ijms26051921	A22	58
R076<br>P00775	PCSK9 Inhibitor Alirocumab Improves Diabetic Cardiomyopathy Through the ERK/p38 MAPK Signaling Pathway.<br>DOI 10.3390/ijms27052341	A26	59

Inventory ID	Publication and DOI	Appraisal record	Manuscript reference
R080<br>P00127	PCSK9 Modulates Macrophage Polarization-Mediated Ventricular Remodeling after Myocardial Infarction.<br>DOI 10.1155/2022/7685796	A27	60
R087<br>P00056	PCSK9 regulates pyroptosis via mtDNA damage in chronic myocardial ischemia.<br>DOI 10.1007/s00395-020-00832-w	B87	61
R105<br>P00333	The comparative effects of high dose atorvastatin and proprotein convertase subtilisin/kexin type 9 inhibitor on the mitochondria of oxidative muscle fibers in obese-insulin resistant female rats.<br>DOI 10.1016/j.taap.2019.114741	B105	62
E10<br>Extra	PCSK9 inhibitor and atorvastatin reduce cardiac impairment in ovariectomized prediabetic rats via improved mitochondrial function and Ca2+ regulation<br>DOI 10.1111/jcmm.15556	B extra	63
R092<br>P05654	Positive correlation of plasma PCSK9 levels with HbA1c in patients with type 2 diabetes.<br>DOI 10.1002/dmrr.2712	B92	64
R100<br>P00283	Serum PCSK9 Correlates with PTX3 and Apolipoproteins B, A1, and C3 Concentrations in Patients with Type 2 Diabetes.<br>DOI 10.1155/2021/7956161	A36	65
R061<br>P02365	Obesity and type 2 diabetes are associated with elevated PCSK9 levels in young women.<br>DOI 10.1111/pedi.12490	A46	66
R091<br>P04283	Plasma proprotein-convertase-subtilisin/kexin type 9 (PCSK9) and cardiovascular events in type 2 diabetes.<br>DOI 10.1111/dom.13181	B91	67
R006<br>P06104	Association between serum PCSK9 and coronary heart disease in patients with type 2 diabetes mellitus.<br>DOI 10.1186/s13098-023-01238-z	A3	68
R014<br>P05068	Circulating PCSK9 levels are not associated with the conversion to type 2 diabetes.<br>DOI 10.1016/j.atherosclerosis.2019.11.027	A6	69
R079<br>P02536	PCSK9 levels in abdominally obese men: association with cardiometabolic risk profile and effects of a one-year lifestyle modification program.<br>DOI 10.1016/j.atherosclerosis.2014.07.010	B79	70
R073<br>P04299	PCSK9 in diabetic kidney disease.<br>DOI 10.1111/eci.12661	B73	71
R047<br>P04198	Increased serum PCSK9 levels are associated with renal function impairment in patients with type 2 diabetes mellitus.<br>DOI 10.1080/0886022x.2023.2215880	A15	72
R009<br>P07056	C679X loss-of-function PCSK9 variant lowers fasting glucose levels in a black South African population: A longitudinal study.<br>DOI 10.1016/j.diabres.2018.09.012	B9	73
R108<br>P02414	The loss-of-function PCSK9 p.R46L genetic variant does not alter glucose homeostasis.<br>DOI 10.1007/s00125-015-3659-8	A39	74

Inventory ID	Publication and DOI	Appraisal record	Manuscript reference
R024<br>P04975	Effect of the PCSK9 R46L genetic variant on plasma insulin and glucose levels, risk of diabetes mellitus and cardiovascular disease: A meta-analysis.<br>DOI 10.1016/j.numecd.2024.04.007	B24	75
R057<br>P07022	Multiomic Mendelian Randomization Study Investigating the Impact of PCSK9 and HMGCR Inhibition on Type 2 Diabetes Across Five Populations.<br>DOI 10.2337/db24-0451	A18	78
R060<br>P06499	No effect of PCSK9 inhibitor alirocumab on the incidence of diabetes in a pooled analysis from 10 ODYSSEY Phase 3 studies.<br>DOI 10.1093/eurheartj/ehw292	A19	80
E04<br>P01499	Efficacy and safety of evolocumab in individuals with type 2 diabetes mellitus: primary results of the randomised controlled BANTING study.<br>DOI 10.1007/s00125-019-4856-7	A59	81
E09<br>P06386	Real-world data on metabolic effects of PCSK9 inhibitors in a tertiary care center in patients with and without diabetes mellitus.<br>DOI 10.1186/s12933-021-01283-w	A64	82
R055<br>P06078	Lower Risk of Type 2 Diabetes Mellitus With PCSK9 Inhibitors Compared With Other Lipid-Lowering Agents Among Patients With Hyperlipidaemia.<br>DOI 10.1002/dmrr.70188	B55	83
R039<br>P06489	Hyperglycaemic disorders associated with PCSK9 inhibitors: a real-world, pharmacovigilance study.<br>DOI 10.1093/eurjpc/zwab209	B39	84
R098<br>P02325	Risk of Incident Diabetes Related to Lipoprotein(a), LDL Cholesterol, and Their Changes With Alirocumab: Post Hoc Analyses of the ODYSSEY OUTCOMES Randomized Trial.<br>DOI 10.2337/dc24-2110	A35	85
E03<br>P06527	Randomised, phase 1, dose-finding study of MEDI4166, a PCSK9 antibody and GLP-1 analogue fusion molecule, in overweight or obese patients with type 2 diabetes mellitus.<br>DOI 10.1007/s00125-018-4789-6	A58	86
R044<br>P00207	Inclisiran inhibits oxidized low-density lipoprotein-induced foam cell formation in Raw264.7 macrophages via activating the PPARγ pathway.<br>DOI 10.1080/08916934.2022.2051142	B44	87
R054<br>P02134	Lipoprotein(a) lowering by alirocumab reduces the total burden of cardiovascular events independent of lowdensity lipoprotein cholesterol lowering: ODYSSEY OUTCOMES trial.<br>DOI 10.1093/eurheartj/ehaa649	A17	88
R112<br>P06656	Triglyceride Levels, Alirocumab Treatment, and Cardiovascular Outcomes After an Acute Coronary Syndrome.<br>DOI 10.1016/j.jacc.2024.06.035	A41	89
R020<br>P01698	Effect of alirocumab on postprandial hyperlipidaemia in patients with type 2 diabetes: A randomized, double-blind, placebo-controlled, cross-over trial.<br>DOI 10.1111/dom.16305	A8	90
R090<br>P01696	Plasma PCSK9 correlates with apoB-48-containing triglyceride-rich lipoprotein production in men with insulin resistance.<br>DOI 10.1194/jlr.m086264	A32	91

Inventory ID	Publication and DOI	Appraisal record	Manuscript reference
R053<br>P01093	LDL subspecies and lipidome change by evolocumab add-on therapy to empagliflozin in patients with type 2 diabetes: A prespecified secondary analysis of a randomized clinical trial.<br>DOI 10.1016/j.atherosclerosis.2026.120686	B53	92
R021<br>P07694	Effect of Empagliflozin with or without the Addition of Evolocumab on HDL Subspecies in Individuals with Type 2 Diabetes Mellitus: A Post Hoc Analysis of the EXCEED-BHS3 Trial.<br>DOI 10.3390/ijms25074108	A9	93

Appraised publications not individually cited

23 publications. Citation status is descriptive and does not imply scientific exclusion.

Inventory ID	Publication and DOI	Appraisal record	Manuscript reference
R003<br>P07006	[The triglyceride-lowering effects of PCSK9 inhibitor differ in patients with different baseline triglyceride levels].<br>DOI 10.12122/j.issn.1673-4254.2020.08.11	A1	—
R004<br>P06497	Alirocumab safety in people with and without diabetes mellitus: pooled data from 14 ODYSSEY trials.<br>DOI 10.1111/dme.13817	A2	—
R016<br>P00183	Coronary Endothelial Dysfunction Is Associated With Elevated Serum PCSK9 Levels in People With HIV Independent of Low-Density Lipoprotein Cholesterol.<br>DOI 10.1161/jaha.118.009996	A7	—
R026<br>P06868	Effects of Alirocumab on Triglyceride Metabolism: A Fat-Tolerance Test and Nuclear Magnetic Resonance Spectroscopy Study.<br>DOI 10.3390/biomedicines10010193	A10	—
R028<br>P07680	Efficacy and Safety of Alirocumab 300 mg Every 4 Weeks in Individuals With Type 2 Diabetes on Maximally Tolerated Statin.<br>DOI 10.1210/jc.2018-02703	A43	—
R029<br>P02295	Efficacy and safety of alirocumab among individuals with diabetes mellitus and atherosclerotic cardiovascular disease in the ODYSSEY phase 3 trials.<br>DOI 10.1111/dom.13384	A11	—
R030<br>P05422	Evaluation of the efficacy, safety and glycaemic effects of evolocumab (AMG 145) in hypercholesterolaemic patients stratified by glycaemic status and metabolic syndrome.<br>DOI 10.1111/dom.12788	B30	—
R033<br>P06823	Familial hypercholesterolemia: PCSK9 InsLEU genetic variant and prediabetes/diabetes risk.<br>DOI 10.1016/j.jacl.2015.08.005	B33	—
R040<br>P00366	Impact of Bariatric Surgery on Subtilisin/Kexin Type 9 (PCSK9) Gene Expression and Inflammation in the Adipose Tissue of Obese Diabetic Rats.<br>DOI 10.3390/ijms242316978	A14	—

Inventory ID	Publication and DOI	Appraisal record	Manuscript reference
R042<br>P00302	Improvement of retinal microvascular function after initiation of lipid-lowering therapy with PCSK9 inhibitors - An observational study.<br>DOI 10.1016/j.jacl.2025.03.006	B42	—
R045<br>P01694	Inclisiran Lowers LDL-C and PCSK9 Irrespective of Diabetes Status: The ORION-1 Randomized Clinical Trial.<br>DOI 10.2337/dc18-1491	A44	—
R050<br>P04851	Lack of a relationship between plasma PCSK9 concentrations and hepatic lipoprotein kinetics in obese people.<br>DOI 10.1016/j.trsl.2011.06.006	A45	—
R051<br>P02429	Lack of association between plasma PCSK9 and LDL-apoB100 catabolism in patients with uncontrolled type 2 diabetes.<br>DOI 10.1016/j.atherosclerosis.2011.07.098	B51	—
R069<br>P04022	PCSK9 deficiency results in a specific shedding of excess LDLR in female mice only: Role of hepatic cholesterol.<br>DOI 10.1016/j.bbalip.2022.159217	A21	—
R082<br>P06768	PCSK9 positively correlates with plasma sdLDL in community-dwelling population but not in diabetic participants after confounder adjustment.<br>DOI 10.1097/md.0000000000015062	A28	—
R084<br>P00141	PCSK9 promotes the secretion of pro-inflammatory cytokines by macrophages to aggravate H/R-induced cardiomyocyte injury via activating NF-κB signalling.<br>DOI 10.4149/gpb_2019057	A47	—
R095<br>P00175	Protective effect of evolocumab on Müller cells in the rat retina under hyperglycaemic and hypoxic conditions.<br>DOI 10.1016/j.jdiacomp.2023.108593	B95	—
R096<br>P02352	Relation of Lipoprotein(a) Levels to Incident Type 2 Diabetes and Modification by Alirocumab Treatment.<br>DOI 10.2337/dc20-2842	A34	—
R101<br>P01709	Serum Proprotein Convertase Subtilisin/Kexin Type 9 (PCSK9) is Independently Associated with Insulin Resistance, Triglycerides, Lipoprotein(a) Levels but not Low-Density Lipoprotein Cholesterol Levels in a General Population.<br>DOI 10.5551/jat.56390	A37	—
R107<br>P07719	The Impact of PCSK9 Inhibitors on Development of Retinal Vascular Occlusions<br>DOI 10.1016/j.ajo.2026.06.038	B107	—
R109<br>P00431	The relationship between oxLDL, sLOX-1, PCSK9 and carotid intima-media thickness in patients with prediabetes and type 2 diabetes.<br>DOI 10.1038/s41598-025-89220-9	A40	—
E06<br>P05333	Evolocumab safety and efficacy in hypercholesterolemia patients with or without diabetes: a retrospective realworld analysis.<br>DOI 10.1186/s13098-025-01587-x	A61	—
E08<br>P01736	Effects of evolocumab in individuals with type 2 diabetes with and without atherogenic dyslipidemia: An analysis from BANTING and BERSON.<br>DOI 10.1186/s12933-021-01287-6	A63	—

Full texts recorded as unavailable

These 15 records belong to the original retrieval list and are outside the 108-publication appraisal corpus. The status follows the final synthesis supplied for this reconciliation; the earlier Excel status alone was not used to determine it. Lack of access is distinct from scientific ineligibility. Abstract-only findings were not treated as decisive full-text evidence.

Retrieval record	Publication	Recorded DOI or PMID
R002<br>P00188	[Knockdown of PCSK9 inhibits endothelial-to-mesenchymal transition of human aortic endothelial cells].	PMID 34906295
R005<br>P00169	Arylesterase activity but not PCSK9 levels is associated with chronic kidney disease in type 2 diabetes.	10.1007/s11255-020-02547-2
R013<br>P03200	Circulating PCSK9 in patients with type 2 diabetes and related metabolic disorders.	10.1016/j.arteri.2015.11.001
R017<br>P00092	Cross-talk between LOX-1 and PCSK9 in vascular tissues.	10.1093/cvr/cvv178
R018<br>P00280	Diagnostic and therapeutic potential of PCSK9 in diabetic tubulopathy: Evidence from observational and experimental studies.	10.1016/j.intimp.2025.115907
R031<br>P05449	Evolocumab in Patients With High-Risk Diabetes: Results From the VESALIUS-CV Trial.	10.2337/dc26-0847
R037<br>P04349	High Serum PCSK9 Is Associated With Increased Risk of New-Onset Diabetes After Transplantation in Renal Transplant Recipients.	10.2337/dc16-2258
R038<br>P08398	Human PCSK9D374Y exacerbates methionine choline deficiency diet-induced nonalcoholic steatohepatitis in mice	10.16352/j.issn.1001-6325.2025.05.0637
R046<br>P00888	Increase of PCSK9 expression in diabetes promotes VEGFR2 ubiquitination to inhibit endothelial function and skin wound healing.	10.1007/s11427-023-2688-8
R064<br>P00094	PCSK9 and LRP5 in macrophage lipid internalization and inflammation.	10.1093/cvr/cvaa254
R067<br>P04803	PCSK9 contributes to diabetes-associated chronic kidney injury beyond LDL cholesterol regulation: genetic and observational evidence.	10.1093/qjmed/hcag126
R070<br>P02395	PCSK9 deficiency results in increased ectopic fat accumulation in experimental models and in humans.	10.1177/2047487317724342
R077<br>P04321	PCSK9 inhibitor effectively attenuates cardiometabolic impairment in obese-insulin resistant rats.	10.1016/j.ejphar.2020.173347
R103<br>P03833	Substantial PCSK9 inactivation in β-cells does not modify glucose homeostasis or insulin secretion in mice.	10.1016/j.bbalip.2021.158968
R104<br>P00378	Sweroside attenuates heart failure with preserved ejection fraction via targeting PCSK9-CD36 interaction to reduce cardiac-hepatic lipotoxicity.	10.1016/j.phymed.2026.158403

Bibliographic caution: the original recovery report flags a problem with the DOI for retrieval record 38. Its title and original identifier are retained here without treating that DOI as validated. The list records access limitations reported in the final synthesis, not a fresh search for availability.

Publication specific decisions made during revision

The following 40 records had no individual citation in the submitted version. The decision is implemented in the revised manuscript. The reason describes the current editorial assessment rather than reconstructing an undocumented historical choice. Related references identify coverage of a narrative point, not necessarily an identical population or endpoint. All reference numbers refer to the revised bibliography.

Inventory ID	Revision decision	Rationale and relationship to cited evidence
R001<br>P01103	Integrated	Adds a directly relevant clinical comparison reporting favourable inflammatory-marker changes in type 2 diabetes. Unequal LDL lowering, limited allocation/blinding information and reporting inconsistencies prevent an LDL-independent inference. Related revised references: 38, 41. Integrated in section 5.3 as reference 39.
R003<br>P07006	Inventory only	Primarily descriptive triglyceride lowering in an uncontrolled cohort with few diabetic participants. Concomitant fibrate/ezetimibe changes and regression to the mean limit attribution. Interventional lipoprotein effects are already represented; the revision also adds R112 for triglycerides and events. Related revised references: 90, 92, 93.
R004<br>P06497	Inventory only	Adds pooled safety detail but does not change the glycaemic-safety conclusion supported by larger trial analyses. ODYSSEY programme overlap limits independent replication. The manuscript does not report this pool's specific adverse-event rates. Related revised references: 2, 80.
R006<br>P06104	Integrated	Adds a large positive prognostic cohort in type 2 diabetes to balance the discordant prospective findings already discussed. Angiography-based selection and residual confounding prevent universal prediction or causal treatment claims. Related revised references: 67. Integrated in section 8.1 as reference 68.
R012<br>P00262	Integrated	Adds human monocyte-subset observations not represented by cell experiments or inclisiran immune analyses. Small samples, statin-conditioned associations, multiple comparisons and compositional percentages restrict inference to an exploratory association. Related revised references: 15, 16, 18. Integrated in section 4.2 as reference 23.
R016<br>P00183	Inventory only	The HIV-focused cross-sectional association has limited applicability to type 2 diabetes. R32 was preferred for the functional vascular bridge because it examines change during treatment, although it too remains uncontrolled and largely non-diabetic. Other vascular associations are already discussed. Related revised references: 32, 67.
R026<br>P06868	Inventory only	An uncontrolled pilot with four diabetic participants adds limited information beyond the randomised postprandial study already cited. Its PCSK9-assay observation would require citation if discussed, but the revised manuscript does not interpret PCSK9 concentrations during antibody treatment. Related revised references: 90.
R028<br>P07680	Inventory only	Adds a particular alirocumab dosing schedule without immune outcomes or a discordant glycaemic signal. Existing references adequately support the clinical conclusion. CHOICE I is not assumed to overlap the 14-trial safety pool; the decision is based on contribution, not presumed duplication. Related revised references: 1, 2, 80, 81.
R029<br>P02295	Inventory only	Overlapping ODYSSEY phase 3 pooling adds lipid efficacy and descriptive glucose stability without a distinct immune mechanism. The review already cites the outcome-trial diabetes analysis and the incident-diabetes pool; it does not count related pools as new independent trials. Related revised references: 2, 80.
R030<br>P05422	Inventory only	DESCARTES adds one-year metabolic measurements but no discordant clinical or immune finding that changes the narrative. Evolocumab glycaemic safety is already represented. The manuscript does not reproduce the specific HOMA results or rates from this report. Related revised references: 1, 81.

Inventory ID	Revision decision	Rationale and relationship to cited evidence
R032 P00189	Integrated	Adds human coronary endothelial function, distinct from plaque or inflammatory imaging. The near absence of diabetes, lack of placebo, baseline selection and concurrent LDL reduction prevent attribution to a direct anti-inflammatory pathway. Related revised references: 34, 35, 36. Integrated in section 5.2 as reference 37.
R033 P06823	Inventory only	A specific InsLEU association in LDLR familial hypercholesterolaemia adds a limited cross-sectional genotype phenotype. Existing genetic references support variant and instrument heterogeneity without attempting an exhaustive catalogue. They are not claimed to reproduce the InsLEU result. Related revised references: 73, 74, 75, 78.
R034 P00068	Integrated	Adds mixed human whole-blood transcriptional findings, including unchanged inflammatory genes and discordant vascular/coagulation signals. The main reported within-treatment analysis is not a formal placebo-adjusted treatment effect, and mRNA is not equivalent to protein function. Related revised references: 18, 38, 41. Integrated in section 5.3 as reference 40.
R040 P00366	Inventory only	Bariatric surgery changes multiple exposures; PCSK9 was not directly perturbed and TNF-alpha protein did not significantly decrease. This would broaden a surgical/adipose digression without resolving the central target-mechanism question. Related adipose and tissue/circulation issues are already discussed. Related revised references: 51, 57.
R042 P00302	Inventory only	The retinal physiological endpoint is peripheral to the review's central question, with only two diabetic participants, no control and statin-subgroup dependence. It has no exact substitute in the bibliography; the decision is one of scope, with vascular translation represented elsewhere and by R32. Related revised references: 34, 35, 36.
R045 P01694	Inventory only	Adds early inclisiran lipid efficacy in small diabetic subgroups. Later ORION pooled evidence and the dedicated ORION-1 immune analysis already support the stated claims. The main report does not supply quantitative inflammatory results sufficient for an additional immune conclusion. Related revised references: 17, 18.
R047 P04198	Integrated	Adds a positive renal biomarker association to balance the cited null cohort. Cross-sectional timing, modest discrimination and covariate selection prevent causal or diagnostic claims and do not demonstrate renal benefit from treatment. Related revised references: 6, 52, 53, 71. Integrated in section 8.1 as reference 72.
R049 P00759	Integrated	Adds a clinically relevant FOURIER inflammatory-disease subgroup in which cardiovascular benefit occurred without hsCRP reduction. Post hoc analysis, subgroup heterogeneity and inconclusive primary-endpoint interaction preclude a claim of immune mediation. Related revised references: 1, 41. Integrated in section 5.3 as reference 42.
R050 P04851	Inventory only	The negative hepatic VLDL-triglyceride kinetic association in non-diabetic obesity addresses a specialised physiological question, not immune response or treatment effect. It is not an exact negative replication of intestinal apoB-48 production in R90. The review avoids a general claim that PCSK9 increases all TRL production. Related revised references: 90, 92, 93.
R051 P02429	Inventory only	The small LDL-apoB100 kinetic study, with very small glycaemic subgroups and unstable modelling, does not assess antibody response. A dedicated clearance discussion is unnecessary for the biomarker limitation stated in the review; no existing source is claimed to reproduce this precise endpoint. Related revised references: 64, 67, 69, 70, 71.
R058 P04063	Integrated	Adds a glucose-responsive ChREBP mechanism supported by promoter and perturbation experiments, distinct from insulin regulation. High experimental exposures and a small uncontrolled human component outside type 2 diabetes limit inference about clinical metformin benefit or PCSK9-targeted glucose lowering. Related revised references: 47, 48, 49, 51. Integrated in section 6.3 as reference 50.

Inventory ID	Revision decision	Rationale and relationship to cited evidence
R059 P00222	Integrated	Adds in vivo atherosclerosis and inflammatory-pathway perturbation without detected plasma-lipid differences. Small molecular samples, control limitations and non-equivalence of lipids require caution; systemic lentiviral silencing is not antibody therapy. Related revised references: 15, 20, 24. Integrated in section 4.1 as reference 21.
R061 P02365	Integrated	Adds young participants and sex-conditioned associations with obesity/type 2 diabetes not represented by the adult or mouse studies. Cross-sectional observations and separate significance by sex do not establish a formal interaction or therapeutic response. Related revised references: 45, 49, 64, 67, 69, 70. Integrated in section 8.1 as reference 66.
R069 P04022	Inventory only	The detailed sex-dependent LDLR-shedding mechanism does not change the main compartment conclusion; key human/antibody details were in an unavailable supplement. The review does not discuss shedding-specific results. General receptor and sex heterogeneity are represented elsewhere and by added R61. Related revised references: 5, 7, 12, 13, 45.
R080 P00127	Integrated	Adds functional immune-compartment perturbation after myocardial infarction, distinct from cardiomyocyte bioenergetics or fibrosis alone. Systemic knockout, non-specific macrophage depletion, absent lipid measurements and short follow-up prevent antibody or chronic-remodelling conclusions. Related revised references: 7, 58, 59. Integrated in section 7.3 as reference 60.
R082 P06768	Inventory only	The adjusted sdLDL association is cross-sectional, with reporting problems and no convincing subgroup interaction. It adds neither a treatment effect nor an immune mechanism. The exact cohort is not replicated by the cited lipidomic studies; the specific association is not used in the narrative. Related revised references: 64, 67, 69, 70, 71, 92.
R084 P00141	Inventory only	The coculture pathway is relevant but has omitted doses, uncertain cytokine source, limited replication and no rescue. Added R80 and R87 provide preferred complementary perturbation evidence for the cardiac immune discussion. They do not replicate the precise conditioned-medium experiments, which are not described in the text. Related revised references: 15, 58.
R087 P00056	Integrated	Adds a cardiomyocyte mtDNA–ROS–inflammasome–pyroptosis pathway with perturbations at several levels. It complements, rather than replaces, intracellular bioenergetics and endothelial pyroptosis. High protein exposures, global knockout and confounded human observations limit clinical translation. Related revised references: 7, 30, 58. Integrated in section 7.3 as reference 61.
R089 P00357	Integrated	Adds a high-glucose microvascular endothelial model with LOX-1 rescue. The small age-imbalanced human comparison and unusually high PCSK9 values do not validate the cellular mechanism. Missing controls and absence of clinical antibody testing remain explicit limitations. Related revised references: 20, 27, 29, 30. Integrated in section 4.4 as reference 31.
R090 P01696	Integrated	Adds isotope-based intestinal apoB-48 production, distinguishing kinetic production from measured postprandial concentrations. The association is observational, male-only and partly assembled from previous kinetic datasets; it does not show a pharmacological or event-mediated effect. Related revised references: 90, 92, 93. Integrated in section 9.4 as reference 91.
R095 P00175	Inventory only	The retinal-cell model would introduce another organ with weak translational support: uncertain rat target recognition, absent isotype/osmotic controls and toxicity at higher exposures. The review does not develop a dedicated retinal mechanism section or claim clinical ocular benefit; no equivalent Müller-cell source is implied. Related revised references: 29, 30.
R096 P02352	Inventory only	The Lp(a)–incident-diabetes topic is addressed through added R98, which provides additional insulin/HOMA and molar Lp(a) analyses from the same parent trial. The reports are not identical and are not counted as independent replications. R96-specific numbers are not reproduced. Related revised references: 2.

Inventory ID	Revision decision	Rationale and relationship to cited evidence
R098 P02325	Integrated	Adds the distinction between globally neutral incident-diabetes treatment results and exploratory, differently directed associations of lipid changes with diabetes. The biomarker changes are not separately randomised; the finding is not evidence that Lp(a) lowering causes diabetes. Related revised references: 2, 80, 88. Integrated in section 9.2 as reference 85.
R100 P00283	Integrated	Adds a direct type 2 diabetes inflammatory correlate, PTX3, and an inverse adjusted HbA1c association to contrast the positive association already cited. Selected poor control, stepwise models and partly previously described clinical data limit prognostic inference. Related revised references: 32, 64, 67. Integrated in section 8.1 as reference 65.
R101 P01709	Inventory only	Adds a community correlation with insulin resistance in a population with a minority of diabetic participants. Treatment-dependent associations do not validate a biomarker or causal mechanism. The incremental role is limited after R61 and R100; their populations and exact endpoints are not claimed to be identical. Related revised references: 64, 69, 70.
R107 P07719	Inventory only	Retinal occlusion is a peripheral clinical endpoint for this focused immunometabolic review. Residual confounding, different achieved LDL and sample discrepancies prevent a new ocular indication. It is not omitted simply because observational; other cohorts are added where they resolve a central gap. Related revised references: 83.
R109 P00431	Inventory only	The reported prediabetes group has a problematic HbA1c classification, with absent multivariable adjustment and unvalidated ROC estimates. Better characterised biomarker/prognostic evidence already supports the discussion, including added R6. The specific carotid thresholds are not used. Related revised references: 32, 64, 67, 69.
R112 P06656	Integrated	Adds an outcome-trial counterpoint: triglycerides identify baseline risk but their change during alirocumab was not associated with fewer events in the reported model. This is not proof against every TRL mechanism and does not provide an independent trial population. Related revised references: 88, 90, 92, 93. Integrated in section 9.4 as reference 89.
E06 P05333	Inventory only	Persistent-user selection, no untreated comparator, treatment changes and an ambiguous incident-diabetes denominator limit incremental causal information. Existing real-world and surveillance sources already illustrate the interpretive problem. The reported percentage is not used as incidence among those at risk. Related revised references: 82, 83, 84.
E08 P01736	Inventory only	Adds pooled lipid efficacy by dyslipidaemia phenotype without a distinct immune endpoint. BANTING is already cited; BERSON adds participants but does not change the interpretive boundary. The review does not reproduce this pool’s specific remnant estimates or count overlapping reports as new trials. Related revised references: 81, 92, 93.

Revision accounting: 68 originally cited + 17 integrated = 85 cited; 40 originally uncited − 17 integrated = 23 inventory-only publications; 85 + 23 = 108 appraised publications. The 15 unavailable full texts remain outside the original appraised corpus. Three targeted revision additions are documented separately below; they do not retrospectively change the original count of 108. One is retrieval record R103, cited at abstract level only.

Targeted additions requested during editorial revision

The following three publications were addressed during targeted revision retrieval. They are additional to the original 108-publication appraisal corpus. Main-text access was verified for Schmidt et al. and, after author-supplied retrieval, for Ference et al. Peyot et al. was verified through its indexed abstract and is used as contextual counterevidence, not decisive full-text evidence. External supplements were not acquired or declared read. Peyot et al. is the original retrieval record R103, which remains listed among the 15 unavailable full texts; citing its abstract does not mean the full text was obtained.

Publication	Revised reference	Access level and interpretation

Publication	Revised reference	Access level and interpretation
Schmidt et al 2017<br>PCSK9 genetic variants and risk of type 2 diabetes: a mendelian randomisation study.<br>DOI 10.1016/s2213-8587(16)30396-5	Reference 76	Main text verified in Europe PMC XML; separate external supplements not read. Target-based genetic analysis. Per 1 mmol/L genetically lower LDL, diabetes OR 1.29 (95% CI 1.11–1.50). Lifelong exposure, European ancestry and incident/prevalent diabetes constrain drug translation. Does not estimate antibody treatment risk.
Ference et al 2016<br>Variation in PCSK9 and HMGCR and Risk of Cardiovascular Disease and Diabetes.<br>DOI 10.1056/nejmoa1604304	Reference 77	Main text verified in the author-supplied publisher PDF NEJMoa1604304.pdf, 10 pages (journal pages 2144–2153), on 17 October 2026. Methods, results, discussion, Table 1 and Figures 1–4 were examined; the separate supplementary appendix was not supplied. The genetic comparison reports cardiovascular protection with higher diabetes odds per unit lower LDL. Its primary diabetes analysis combines prevalent and incident cases; incident-diabetes analyses and fasting-glucose subgroups must be distinguished. Lifelong exposure and extracellular-versus-genetic differences prevent direct translation to antibody treatment risk.
Peyot et al 2021<br>Substantial PCSK9 inactivation in β-cells does not modify glucose homeostasis or insulin secretion in mice.<br>DOI 10.1016/j.bbalip.2021.158968	Reference 19	Abstract verified; full text not recovered. Original record R103 P03833. Reported preserved glucose tolerance and insulin secretion after substantial beta-cell PCSK9 inactivation. Contextual counterevidence to a uniform pancreatic injury model. Abstract-only access prevents assessment of experimental methods, power and all tested conditions; it does not establish treatment safety.

Current bibliography accounting: 85 cited publications from the original corpus + 3 editorial evidence additions + 4 contextual reviews + 2 peer-review additions = 94 references. The original corpus still comprises 85 cited and 23 inventory-only publications. Among the three editorial evidence additions, two have verified main texts and Peyot et al. remains abstract-only. The two peer-review additions have the explicitly limited verification levels recorded below. Main-text availability and citation status are distinct fields. No targeted addition has been retrospectively inserted into the original 108-publication denominator.

Ference main text verification

The uploaded publisher PDF was read in full. The 14-study individual-level analysis used seven PCSK9 variants and six HMGCR variants, age- and sex-adjusted logistic models, LDL-scaled estimates and study-level pooling. The primary cardiovascular endpoint was myocardial infarction or coronary death. The primary diabetes analysis combined 4340 prevalent and 6295 incident cases; it is not an incident-only treatment trial. Methods and sample definitions are on journal pages 2145–2146; outcome results and Figures 1–4 are on pages 2147–2151.

Figure 3 gives the PCSK9 diabetes OR of 1.11 (95% CI 1.04–1.19) per 10 mg/dL genetically lower LDL for the combined diabetes analysis. Figure 4 separately reports the incident-only estimate, 1.11 (1.00–1.26), and the fasting-glucose subgroups: impaired fasting glucose 1.22 (1.03–1.45), normal fasting glucose 0.99 (0.84–1.17). Subgroup significance alone does not establish interaction. The discussion on page 2152 explicitly recognises lifelong exposure and the potential biological difference between extracellular antibody binding and genetic PCSK9 perturbation. Detailed analyses delegated to the unavailable supplementary appendix are not independently certified here.

Contextual reviews used to position the contribution

Four contextual reviews were examined during the originality revision on 17 October 2026. They provide context rather than additional primary observations or independent cohorts and remain outside the historical 108-publication corpus. Current accounting is 85 original-corpus citations + 3 editorial evidence additions + 4 contextual reviews + 2 peer-review additions =

94 references. The original corpus remains 85 cited + 23 inventory-only = 108 publications. The 15 unavailable original main texts and the abstract-only status of Peyot et al. are unchanged.

Reference 8 Päth et al. 2022

Päth, G.; Perakakis, N.; Mantzoros, C.S.; Seufert, J. PCSK9 inhibition and cholesterol homeostasis in insulin producing β-cells. Lipids Health Dis. 2022, 21, 138. https://doi.org/10.1186/s12944-022-01751-6.

Access: main text examined in Europe PMC full-text XML, PMC9756761. External supplementary files were not appraised. Existing scope: β-cell cholesterol handling, knockout models, genetic variants and inhibitor-treated patients; already distinguishes local islet biology from circulating PCSK9. Contribution addressed in the revised manuscript: Extends that compartment distinction to immune and platelet responses and to renal and cardiac counterevidence; tests whether the experimental perturbation models the treatment.

Reference 9 Lu et al. 2023

Lu, F.; Li, E.; Yang, X. The association between circulatory, local pancreatic PCSK9 and type 2 diabetes mellitus: The effects of antidiabetic drugs on PCSK9. Heliyon 2023, 9, e19371. https://doi.org/10.1016/j.heliyon.2023.e19371.

Access: main text examined in Europe PMC full-text XML, PMC10558357. External supplementary files were not appraised. Existing scope: Circulating and pancreatic PCSK9, conflicting islet findings, new-onset diabetes and glucose-lowering or natural agents that modify PCSK9. Contribution addressed in the revised manuscript: Keeps changes in PCSK9 during glucose-lowering therapy separate from evidence that PCSK9 inhibition itself lowers glucose; interprets pancreatic findings alongside tissue-specific effects elsewhere.

Reference 10 Katsuki et al. 2024

Katsuki, S.; Jha, P.K.; Aikawa, E.; Aikawa, M. The role of proprotein convertase subtilisin/kexin 9 (PCSK9) in macrophage activation: a focus on its LDL receptor-independent mechanisms. Front. Cardiovasc. Med. 2024, 11, 1431398. https://doi.org/10.3389/fcvm.2024.1431398.

Access: main text examined in the publisher full-text HTML. External supplementary files were not appraised. Existing scope: Macrophage activation through lipid-dependent, LDLR-dependent and LDLR-independent pathways; acknowledges uncertainty about clinical anti-inflammatory effects. Contribution addressed in the revised manuscript: Applies those mechanistic distinctions to type 2 diabetes across cell and organ systems and asks separately whether a pathway contributes to clinical event reduction.

Reference 11 Circulatory inflammation review 2026

Deng, B.-Y.; Wang, J.-Y.; Yuan, Y.-J.; Dong, X.-Y.; Lai, H.-M.; Peng, H.; Yang, Y.-N.; Yu, X.-L. The Impact of PCSK9 Inhibitors on Circulatory Inflammation: Mechanisms, Evidence, and Application Prospects. JACC Asia 2026, 6, 1323–1339. https://doi.org/10.1016/j.jacasi.2026.05.033.

Access: main text examined in Europe PMC full-text XML, PMC13458932. External supplementary files were not appraised. Existing scope: Distinguishes direct immunomodulation, lipid-mediated plaque remodelling and systemic biomarkers; includes multisystem findings and translational cautions. Contribution addressed in the revised manuscript: Shares that caution. The present emphasis is the diabetes-specific reconciliation of genetic glycaemic signals, pancreatic counterevidence, organ-specific PCSK9 loss and the reach of antibodies versus hepatic silencing.

Targeted additions during external peer review

These two publications were added on 23 October 2026 in response to reviewer comments. Their citation does not enlarge the historical 108-publication appraisal corpus. Abstract-level verification is reported as such; neither publication is represented as newly read in full in this revision.

Montalescot et al. 2026 AMUNDSEN

Manuscript reference 79. DOI 10.1001/jama.2026.17302. Indexed and publisher abstracts checked. The reported one-year clinical result was used with its endpoint and follow-up limits. Full main text and separate supplement were not appraised.

Bohula et al. 2018 FOURIER inflammatory-risk analysis

Manuscript reference 94. DOI 10.1161/CIRCULATIONAHA.118.034032. Indexed abstract checked. Used for the reported hsCRP and cardiovascular-risk findings. Full main text and separate supplement were not appraised. This is a secondary analysis of FOURIER, not an independent trial.

Reference numbering crosswalk for this revision

All reference numbers in this inventory have been updated to match first appearance in the current manuscript, including citations within tables. The original source-record identifiers, appraisal identifiers and 40 revision decisions have been preserved. A CSV crosswalk accompanying this supplement maps the 92 references in the pre-peer-review manuscript to their current numbers and identifies the two newly added references.
