# Supplementary material for: PCSK9 in the Immunometabolism of Type 2 Diabetes
Source: Int J Mol Sci. 2026 Oct 29;27(21):9723. doi: 10.3390/ijms27219723 (PMC13763235; doi:10.3390/ijms27219723)
Supplement: Supplementary file 1 [file ijms-27-09723-s001.zip › PCSK9_Supplementary_S3_Figure_Preparation.pdf]

Figure preparation and AI transparency

Supplementary File S3
Manuscript ijms-4662068
PCSK9 in the Immunometabolism of Type 2 Diabetes

Scope and available provenance

Earlier submitted versions of Figure 1 and the graphical abstract were produced using OpenAI image generation following author-directed requests. The original technical prompts and an exact model/version identifier were not retained. A complete historical prompt record cannot therefore be supplied. No prompt has been retrospectively reconstructed and presented as an original generation instruction.

For the present revision, both illustrations have been rebuilt as deterministic vector schematics from geometric primitives using Python. ChatGPT in the Codex environment assisted with the code and design specifications. This remains AI-assisted preparation, which is disclosed in the manuscript. The final artwork does not embed the earlier generated raster images or import third-party figure assets. Scientific concepts are grounded in the studies cited in the manuscript; the drawings are conceptual rather than experimental data.

Figure 1 design specification

Panel A identifies mainly liver-derived circulating PCSK9 and extracellular exposure. Panel B depicts intracellular PCSK9 exclusively as symbols inside closed membrane-bound cisternae and a vesicle; no PCSK9 symbol is placed freely in the cytosol. Panel C depicts secretion followed by potential extracellular autocrine or paracrine action. Colours distinguish conceptual routes, not different chemical forms. Separate immune-cell, platelet, liver, pancreas, heart and kidney motifs identify contexts without organ-to-organ causal arrows. The heart and kidney are not connected. The figure does not locate PCSK9 protein within mitochondria or assign one direction of effect across all tissues.

Graphical abstract design specification

The graphical abstract separates compartments, experimental biology and clinical interpretation. The intracellular PCSK9 symbols are enclosed within membrane-bound compartments. The statement “Beyond LDL-C lowering” replaces “beyond plasma LDL”. Cardiovascular benefit with antibodies is distinguished from an additional benefit independent of lipoprotein lowering, which remains unproven. Organ and cellular symbols are conceptual and not to scale.

Reproducible source and production files

PCSK9_Illustration_Source.py accompanies this document in the supplementary archive. It reproduces Figure 1 and the graphical abstract with editable text and geometry. Dependencies are Python 3, NumPy, Matplotlib, PyMuPDF and Pillow. Run: python PCSK9_Illustration_Source.py output_directory. The script saves vector SVG and PDF files and 600-dpi PNG and TIFF versions. The source contains the actual design implementation used for this revision; it is not an image-generation prompt log.

Verification and responsibility

The revised schematics were inspected for wording, spelling, compartment placement, connector endpoints and legibility. Exported raster dimensions and vector text were checked. The illustrations contain no patient information, research micrographs or newly generated numerical results. The authors remain responsible for scientific accuracy, attribution and the final publication content. The absence of imported artwork assets is a description of this workflow, not a legal guarantee about copyright in AI-generated material.

MDPI policy consulted on 23 October 2026: https://www.mdpi.com/ethics, Artificial Intelligence and Intellectual Property sections. AI use is disclosed in the methods and acknowledgments of the revised manuscript.
